# The *Legionella* IcmSW Complex Directly Interacts with DotL to Mediate Translocation of Adaptor-Dependent Substrates

**DOI:** 10.1371/journal.ppat.1002910

**Published:** 2012-09-13

**Authors:** Molly C. Sutherland, Thuy Linh Nguyen, Victor Tseng, Joseph P. Vogel

**Affiliations:** 1 Department of Molecular Microbiology, Washington University School of Medicine, St. Louis, Missouri, United States of America; 2 New York Medical College, Valhalla, New York, United States of America; Tufts University School of Medicine, United States of America

## Abstract

*Legionella pneumophila* is a Gram-negative bacterium that replicates within human alveolar macrophages by evasion of the host endocytic pathway through the formation of a replicative vacuole. Generation of this vacuole is dependent upon the secretion of over 275 effector proteins into the host cell via the Dot/Icm type IVB secretion system (T4SS). The type IV coupling protein (T4CP) subcomplex, consisting of DotL, DotM, DotN, IcmS and IcmW, was recently defined. DotL is proposed to be the T4CP of the *L. pneumophila* T4SS based on its homology to known T4CPs, which function as inner-membrane receptors for substrates. As a result, DotL is hypothesized to play an integral role(s) in the *L. pneumophila* T4SS for the engagement and translocation of substrates. To elucidate this role, a genetic approach was taken to screen for *dotL* mutants that were unable to survive inside host cells. One mutant, *dotL*Y725Stop, did not interact with the type IV adaptor proteins IcmS/IcmW (IcmSW) leading to the identification of an IcmSW-binding domain on DotL. Interestingly, the *dotL*Y725Stop mutant was competent for export of one class of secreted effectors, the IcmSW-independent substrates, but exhibited a specific defect in secretion of IcmSW-dependent substrates. This differential secretion illustrates that DotL requires a direct interaction with the type IV adaptor proteins for the secretion of a major class of substrates. Thus, by identifying a new target for IcmSW, we have discovered that the type IV adaptors perform an additional role in the export of substrates by the *L. pneumophila* Dot/Icm T4SS.

## Author Summary

Many pathogens are able to survive and grow within eukaryotic host cells. One such pathogen, *Legionella pneumophila*, is able to replicate within macrophages, resulting in a form of pneumonia called Legionnaires' Disease. One key to *L. pneumophila*'s capacity to cause disease is its ability to translocate several hundred proteins into the host cell. These proteins, typically referred to as “effectors”, function to alter the host cell to create a hospitable environment for the bacteria. *L. pneumophila* effectors are exported by a specialized export apparatus, which is encoded by the *dot/icm* genes. However, the mechanism of secretion for these substrates is poorly understood. It is known that a subset of these effectors requires assistance from the type IV adaptor proteins IcmS and IcmW for transport out of the bacterium. It has been shown that IcmSW binds adaptor-dependent secreted proteins in the bacterial cytoplasm prior to their export. Here we report that DotL, an inner membrane component of the Dot/Icm secretion system, also binds IcmSW and this interaction is required for the export of adaptor-dependent substrates. This defines a new role for the type IV adaptors IcmSW and furthers our understanding of how *Legionella* exports substrates into its host cell.

## Introduction

*Legionella pneumophila* is a ubiquitous Gram-negative bacterium that is able to survive and replicate in freshwater protozoa and in human alveolar macrophages, where its proliferation can result in a severe pneumonia known as Legionnaires' Disease [1]–[3]. Following entry into the host cell, the *Legionella* containing vacuole (LCV) evades the host endocytic pathway and avoids fusion with the bactericidal lysosomes. Concurrently, it forms a protective niche by recruitment of endoplasmic reticulum-derived vesicles, including associated markers such as Sec22b and Rab1 (reviewed in [4], [5]).

Intracellular replication of *L. pneumophila* is dependent on the translocation of at least two hundred seventy-five effector proteins into the host cell [6]–[11]. Deciphering the specific functions of individual substrates has been hampered by the lack of detectable homology of many of the effectors to known toxins and the apparent functional redundancy among the substrates [4], [5]. Nevertheless, the mechanisms of action for some substrates have been determined and they include activities such as recruitment and activation of Arf1 and Rab1 at the LCV, subsequent inactivation of Rab1, prevention of LCV acidification, up-regulation of the NF-κB pathway, interference with host cell pro-apoptotic pathways among others (reviewed in [5], [12]).

Secretion of these proteins is dependent upon a large class of genes called *dot* (defect in organelle trafficking) and *icm* (intracellular multiplication defect) [13], [14]. The *dot/icm* genes encode a large, membrane-spanning apparatus that is classified as a type IVB secretion system (T4SS) [15], [16]. The Dot/Icm T4SS is made up of twenty-seven proteins that include two large subcomplexes [17], [18]. The first is called the transmembrane subcomplex and includes the inner membrane proteins DotF(IcmG) and DotG(IcmE), the presumed outer membrane pore DotH(IcmK) and two associated outer membrane lipoproteins DotC and DotD [17]. The second sub-assembly, the DotL T4CP subcomplex, is made up of DotL(IcmO), DotM(IcmP), DotN(IcmJ), IcmS and IcmW [18].

DotL is of particular interest because it has been proposed to function as the type IV coupling protein (T4CP) for the *Legionella* Dot/Icm secretion system [19]. T4CPs are inner membrane components of T4SSs, they are known to interact with substrates prior to their secretion and with other membrane proteins of the T4SSs, thus “coupling” substrates to the T4SS apparatus (reviewed in [20]). In addition, they contain a Walker box motif for ATP hydrolysis and are believed to provide energy to actively drive the export of substrates across the inner and outer membranes of the bacterial cell wall.

The DotL T4CP subcomplex includes four other proteins in addition to DotL. Although DotM and DotN lack homology to well conserved T4SS components, they are known to associate with the inner membrane and are required to stabilize DotL [17]. In contrast, more is known about IcmS and IcmW (IcmSW). These are two small, acidic proteins that interact to form a heterodimer pair and have been called type IV adaptors [18], [21]. They have been shown to bind to a subset of Dot/Icm substrates and are required for their export [22]–[28]. Based on these properties, IcmSW appear to function similarly to the well-characterized T3SS specialized secretion chaperones.

Based on DotL's similarity to T4CPs, we hypothesized that DotL performs an integral role in substrate secretion. To determine DotL's role(s) in effector translocation, a collection of *dotL* mutants that were defective for the intracellular growth of *L. pneumophila* were isolated. Characterization of one *dotL* mutant elucidated a specific interaction with one component of the Dot/Icm T4SS and revealed novel information about the differential mechanism of secretion of IcmSW-dependent and IcmSW-independent substrates by the *L. pneumophila* type IV secretion system.

## Results

### Isolation of DotL mutants with an intracellular growth defect

To ascertain DotL's role in the export of *L. pneumophila* T4SS substrates, we isolated a collection of *dotL* mutants that were unable to replicate inside *Acanthamoeba castellanii*. However, this was challenging as an easy and unbiased method to select for *dot/icm* mutants that are replication incompetent within host cells did not currently exist. In addition, the most common null mutants consist of proteins that are not stable, which would not be useful for structure-function analysis. Therefore, to eliminate unstable proteins we exploited a unique property of *dotL*, known as Δ*dotL* lethality, that exists in the *Legionella* strain Lp02 [19]. In this strain background, *dotL* is not only essential for growth within host cells but, unlike most other *dot/icm* genes, is also required for growth on bacteriologic media [19], [29]. Since these traits are not linked, it was possible to first select for clones from an error-prone PCR generated *dotL* library that were able to complement Δ*dotL* lethality, thus ensuring that they made stable, functional DotL protein. Subsequently, the library was screened for clones that failed to complement a *L. pneumophila* Δ*dotL* mutant for growth within amoebae.

**Figure 1:**
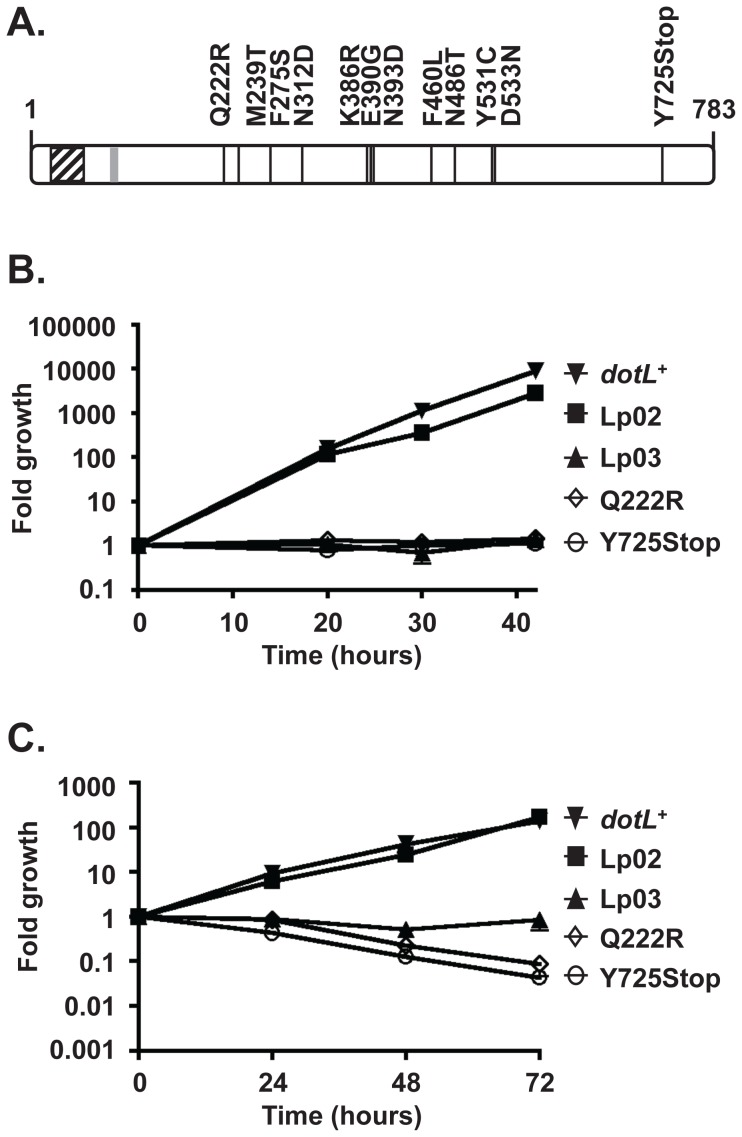
*dotL* mutants are defective for intracellular growth. (A) Schematic of the *dotL* mutants. Twelve *dotL* mutants, each containing a single amino acid change, were identified. A black line indicates their individual locations within the 783 amino acid protein DotL and the residue changed is shown. The DotL transmembrane domains are shown by a hatched box and the Walker A box motif (GSTGSGKT) is shown by a gray box. *A. castellanii* (B) and A/J mouse bone marrow derived macrophages (C) were infected with a wild-type strain (Lp02) (filled squares), a T4SS-deficient strain (Lp03) (filled triangles), a Δ*dotL* strain containing a *dotL^+^* complementing clone (filled inverted triangles) and two representative *dotL* mutants (Q222R (open diamonds) and Y725Stop (open circles)). Fold growth was calculated by dividing the average colony forming units (CFUs) of triplicate wells at a given time point by the average CFUs at time zero. Error bars represent the standard deviation from the mean and the results are representative of three independent experiments.

Based on this strategy, ∼100 *dotL* mutants with an intracellular growth defect were identified by a visual screen from a pool of 3800 plasmids. To eliminate clones with multiple mutations, the plasmids were sequenced revealing twelve *dotL* mutants that contained a single amino acid change (Fig. 1A). The mutants were distributed throughout the protein but none were identified in either the amino-terminal transmembrane domain of DotL or in the canonical Walker A box motif at amino acids 133–140 of DotL (GSTGSGKT) [19], [30]. Eleven of the mutants contained a single amino acid change, while one resulted in a truncation of the C-terminus of DotL at amino acid 725 due to the insertion of a nonsense mutation (Fig. 1A).

In order to validate our screen, the twelve *dotL* mutants were assayed for their ability to complement Δ*dotL* lethality and to express normal levels of DotL protein. Similar to a strain expressing wild-type *dotL*, each of the *dotL* mutants was able to restore viability to a Δ*dotL* mutant when grown on plates (Fig. S1A). Likewise, each of the mutants restored DotL proteins levels in a Δ*dotL* mutant to that observed with wild-type *dotL* (Fig. S1B). In addition, we confirmed that expression of another Dot/Icm component, DotM, was not affected as DotL and DotM require each other for their own stability [17], [18] (Fig. S1B). Therefore, based on these criteria, we conclude that our strategy successfully identified *dotL* mutants with an intracellular growth defect and that their attenuated growth phenotype was not due to reduced DotL or DotM levels.

### DotL plays a conserved role in intracellular growth

The *dotL* mutants were originally identified as being defective for intracellular growth by a visual assessment at forty-eight hours. To measure the extent of their growth defect more precisely, the mutants were assayed for intracellular replication within *A. castellanii* by a quantitative growth curve. In this assay, both the wild-type strain Lp02 and a Δ*dotL* mutant expressing *dotL* ^+^ from a plasmid grew approximately 3,000-fold over forty-two hours (Fig. 1B, filled squares and inverted triangles). In contrast, a strain with a mutation in *dotA* that renders the T4SS non-functional (Lp03), was defective for intracellular growth (Fig. 1B, filled triangles). Similarly, none of the *dotL* mutants could complement the Δ*dotL* mutant for replication in *A. castellanii*, further validating our screen conditions (two representative mutants are shown in Fig. 1B and the remaining are shown in Fig. S2A–B).

To examine if the *dotL* mutants were defective in other hosts, their ability to grow and replicate was assayed in mouse A/J bone marrow derived macrophages (BMM) [31] and in the human monocytic cell line U937 [32]. Similar to amoebae, the wild-type strain Lp02 grew robustly in BMMs and in U937 cells, whereas the T4SS-deficient strain Lp03 was unable to grow in either (Fig. 1C and data not shown). Likewise, all twelve of the *dotL* mutants exhibited a dramatic defect in their ability to replicate within BMM cells (Fig. 1C and Fig. S2C–D) and were attenuated for growth within U937 cells (data not shown). Therefore, the intracellular growth defect of the *dotL* mutants was not restricted to amoebae and occurred in a variety of host cells.

### *dotL* mutants' interaction with other components of the Dot/Icm T4SS

**Figure 2:**
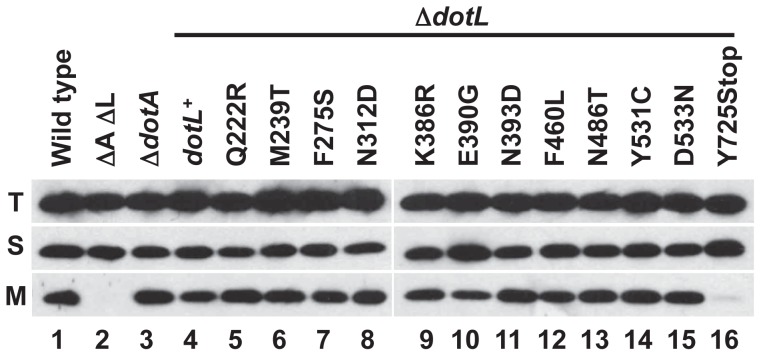
Recruitment of IcmSW to the membrane by the *dotL* mutants. Subcellular localization of IcmS was assayed using *L. pneumophila* lysates separated into total (T), soluble (S) and membrane (M) fractions. Δ*A* Δ*L* is the Δ*dotA* Δ*dotL* double mutant and all of the *dotL* mutants were expressed in a Δ*dotL* background. Protein fractions were analyzed by western blot using an IcmS-specific antibody. Fractions were loaded proportionally and are representative of three independent experiments.

To identify the underlying molecular mechanism(s) for the *dotL* mutants' intracellular growth defect, their ability to assemble the DotL T4CP subcomplex was assessed. One novel feature of this subcomplex is the recruitment of the type IV adaptors IcmSW to the membrane, which can be assayed by membrane fractionation. As previously observed [18], a significant proportion of the total cellular pool of IcmSW is targeted to the membrane in a wild-type strain, while a strain lacking *dotL* is defective for IcmSW-membrane localization (Fig. 2, lanes 1–2). Measuring IcmSW recruitment in a strain lacking *dotL* had to be assessed in a suppressed background, such as Δ*dotA*, due to Δ*dotL* lethality [19], although the absence of *dotA* had no effect on targeting of IcmSW (Fig. 2, lane 3). In contrast to a strain lacking *dotL*, strains containing the *dotL^+^* complementing clone or the majority of the *dotL* mutants were able to recruit IcmSW to the membrane (Fig. 2, lanes 4, 5–15). Strikingly, the *dotL*Y725Stop mutant was unable to recruit IcmSW to the membrane (Fig. 2, lane 16), similar to the defect observed in the Δ*dotL* strain. Proper fractionation of the samples was confirmed by localization of the cytoplasmic protein isocitrate dehydrogenase (ICDH) and the inner membrane protease LepB (Fig. S3). In conclusion, only the *dotL*Y725Stop mutant was defective for recruiting IcmSW to the inner membrane implying that the underlying cause of this mutant's intracellular growth defect may be due to the lack of interaction with the type IV adaptors.

### Functionality of the *dotL*Y725Stop mutant's type IV secretion system

In order to further characterize the role of the C-terminus of DotL, the *dotL*Y725Stop mutant was assayed for two traits that reflect the presence of an assembled and functional Dot/Icm T4SS: contact-dependent cytotoxicity and plasmid transfer. Contact-dependent cytotoxicity is observed when macrophages are exposed to a vast excess of *L. pneumophila* bacteria [33], resulting in pores being formed in the host cell membrane due to caspase-1 mediated cell death triggered by flagellin leakage through an intact T4SS (reviewed in [34], [35]). In this assay, the wild-type strain Lp02 was competent to permeabilize the macrophages whereas Lp03 exhibited greatly reduced levels of permeabilization (Fig. S4). A Δ*dotL* strain complemented with wild-type *dotL* (*dotL* ^+^) or the *dotL*Y725Stop mutant behaved similar to Lp02 (Fig. S4), indicating that the mutant is competent for contact-dependent cytotoxicity and forms a functional apparatus. Similarly, both the wild-type *L. pneumophila* strain and a strain expressing only the *dotL*Y725Stop mutant were competent to transfer an *oriT* ^+^ RSF1010 plasmid to *E. coli* (Table S1), another Dot/Icm dependent phenotype [13], [14]. Consistent with the absence of a phenotype for the *dotL*Y725Stop mutant, both contact dependent cytotoxicity and plasmid mobilization are not dependent on the type IV adaptor IcmS [22], [36] (Table S1). These assays establish that the *dotL*Y725Stop mutant's intracellular growth defect is not due to a failure to assemble a functional T4SS, but is more likely related to its inability to bind IcmSW.

### Identification of the IcmS/IcmW binding domain on DotL

**Figure 3:**
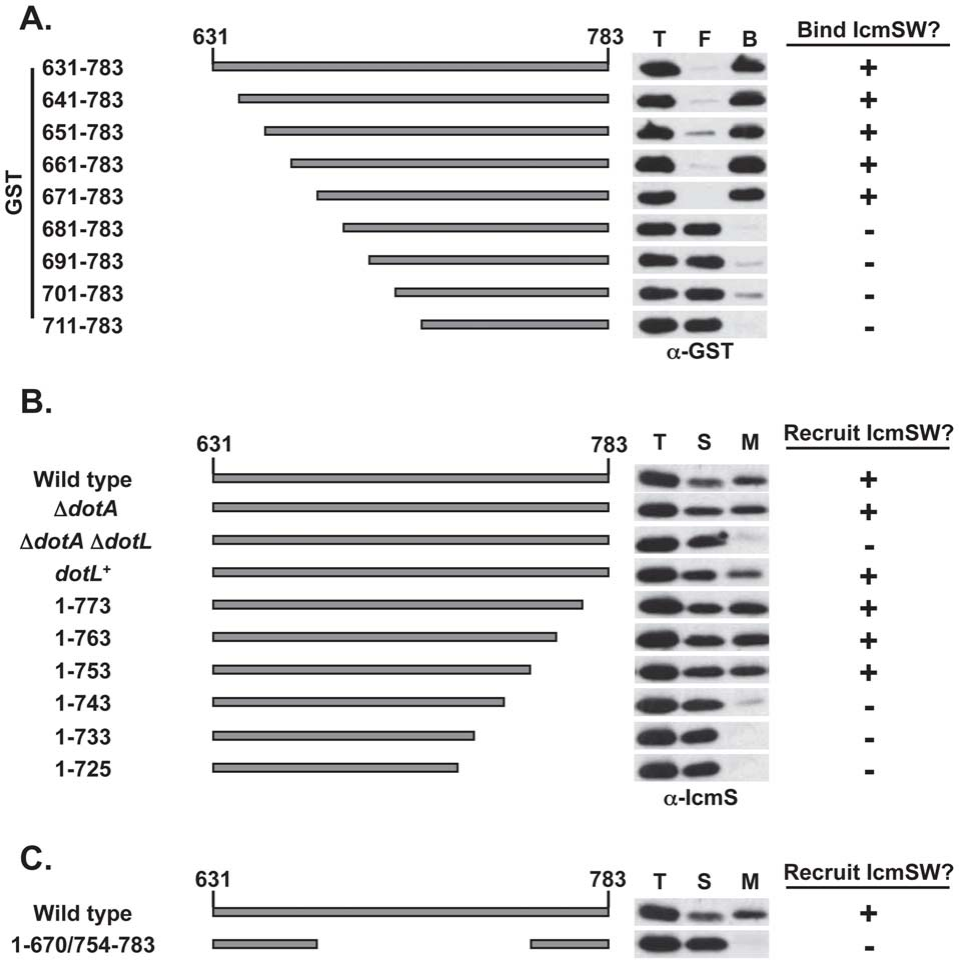
Identification of the IcmSW-binding domain on DotL. (A) *E. coli* lysates co-expressing His:SW and various GST:DotL fragments truncated at their amino terminus were bound to a Ni-NTA column. The presence of the indicated GST:DotL fusions was assessed in the total (T), flow-through (F), and bound fractions (B) in a GST western. A schematic of the C-terminal amino acids 631–783 of DotL illustrates the indicated deletions. (B) *L. pneumophila* lysates expressing C-terminal deletions of DotL were separated into total (T), soluble (S) and membrane (M) fractions. Membrane recruitment of IcmS was determined by western blot using an IcmS-specific antibody. (C) IcmS recruitment was assayed for a *dotL* mutant containing a deletion of the putative IcmSW-binding domain on DotL (amino acids 671–753). Results are representative of three independent experiments.

To further examine the relationship between these proteins, the IcmSW-binding region of DotL was mapped by defining the N-terminal and C-terminal boundaries of the domain followed by a “necessary & sufficiency” test. The N-terminal boundary of the IcmSW-binding domain of DotL was determined by analyzing interactions between histidine tagged-IcmS/IcmW (His:SW) and a series of DotL truncations fused to glutathione S-transferase (GST:DotL). The His:SW heterodimer was recovered with nickel-nitrilotriacetic acid (Ni-NTA) resin and interaction with the GST:DotL fragments was monitored by immunoblot with a GST-specific antibody. In this analysis, the minimal DotL fragment that could bind IcmSW consisted of 113 amino acids from residues 671–783 of DotL (Fig. 3A). The interactions were specific as none of the GST:DotL fragments were recovered from reactions containing the empty His vector (Fig. S5A) and the failure to see an interaction was not due to an inability to bind the Ni-NTA resin as His:SW was recoverable in all reactions (Fig. S5B).

The C-terminal boundary of the IcmSW-binding domain of DotL was determined by assaying recruitment of IcmSW to the membrane as described in Figure 2. Truncations of 10 to 30 amino acids did not affect IcmSW localization to the membrane (Fig. 3B with representative controls in Fig. S5C). In contrast, removal of the last 40 or 50 amino acids of DotL resulted in vastly diminished recruitment of IcmSW (Fig. 3B), although these constructs did express slightly decreased levels of DotL (Fig. S5D), which could be partially responsible for their defect. Based on the above analysis, the N-terminal boundary of the IcmSW-binding domain on DotL begins around amino acid 671 and ends near amino acid 753. Deletion of this domain from wild-type DotL (removal of amino acids 671 to 753) resulted in no IcmSW recruitment to the membrane (Fig. 3C), thus validating the mapped boundaries in Figures 3A and 3B.

**Figure 4:**
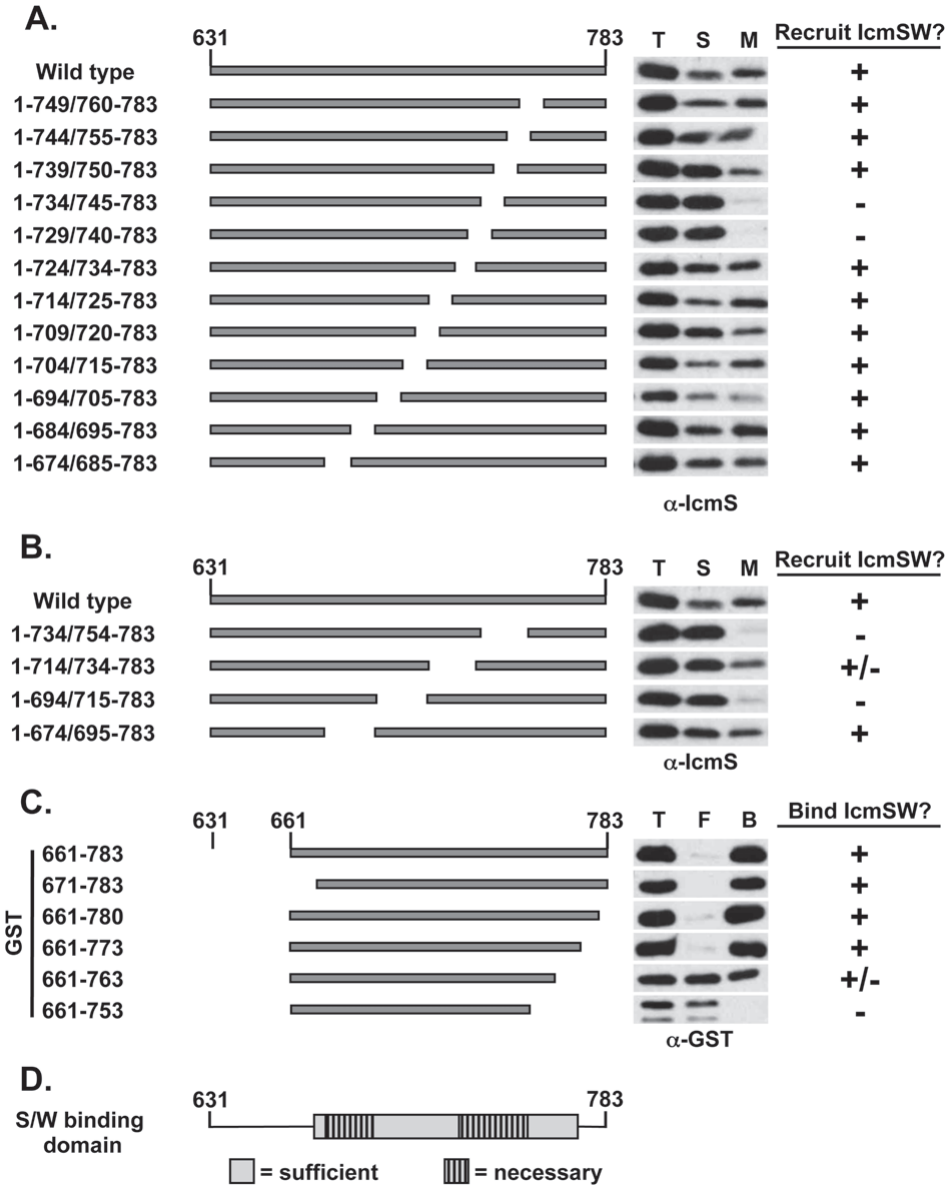
Identification of the domain(s) of DotL that is necessary and sufficient to bind IcmSW. DotL domains necessary for binding IcmSW were identified by assaying *L. pneumophila* strains expressing 10 amino acid (A) and 20 amino acid (B) internal deletions of DotL. Membrane recruitment of IcmS was determined by western blot using an IcmS-specific antibody against total (T), soluble (S) and membrane (M) fractions. (C) The DotL domain sufficient to bind IcmSW was identified by binding lysates co-expressing His:SW and various GST:DotL fragments to a Ni-NTA column. The presence of GST:DotL in the total (T), flow-through (F), and bound fractions (B) was determined by western blotting using a GST-specific antibody. Results are representative of three independent experiments. (D) A schematic of DotL containing amino acids 631–783 is shown to illustrate the properties of the DotL IcmSW-binding domain. The sufficient domain is indicated by a gray box and the necessary domains are denoted by striped boxes.

To further refine the binding domain, a necessary and sufficiency test was performed. A series of internal ten amino acid deletions were constructed throughout the mapped boundaries in full-length DotL. Only two deletions, one removing amino acids 730–739 and the other deleting amino acids 735–744, affected IcmSW binding (Fig. 4A), thus potentially identifying one domain necessary for the interaction between DotL and the adaptors. Since these deletions may affect the fold of this domain, we cannot conclude that DotL binds IcmSW directly via these residues. To further refine the binding domain(s), a set of larger deletions consisting of twenty amino acids revealed a potential second binding domain (Fig. 4B). The first deletion, spanning amino acids 735–753, overlapped the domain identified with the smaller deletions. The second deletion, spanning amino acids 695–714, was located twenty amino acids closer to the N-terminus. Thus, there appears to be at least two critical domains within this region of DotL that are necessary for IcmSW recruitment to the membrane.

Finally, we attempted to identify the minimal fragment of DotL sufficient to interact with IcmSW. The minimal fragment identified in Fig. 3A consisted of 113 amino acids from residues 671 to 783. Although small deletions from the C-terminus of this fragment could bind IcmSW, larger deletions were unstable (Fig. S6A). Therefore, a series of truncations were constructed using a slightly larger DotL fragment containing amino acids 661–783. Removal of 3 or 10 amino acids from the C-terminus of this fragment had no effect, whereas a deletion of 20 amino acids was partially defective and removal of the last 30 amino acids completely prevented DotL binding to IcmSW (Fig. 4C with controls in Fig. S6B–C). In summary, two DotL fragments consisting of 113 amino acids (671–783 or 661–773) were both fully sufficient to interact with IcmSW. Taken as a whole, we propose the minimal domain of DotL that is sufficient to interact with IcmSW likely consists of amino acids 671–773 and this includes two regions (695–714 and 730–753) that are critically important for the interaction (schematic shown in Fig. 4D).

### Direct interaction between DotL and IcmS/IcmW

**Figure 5:**
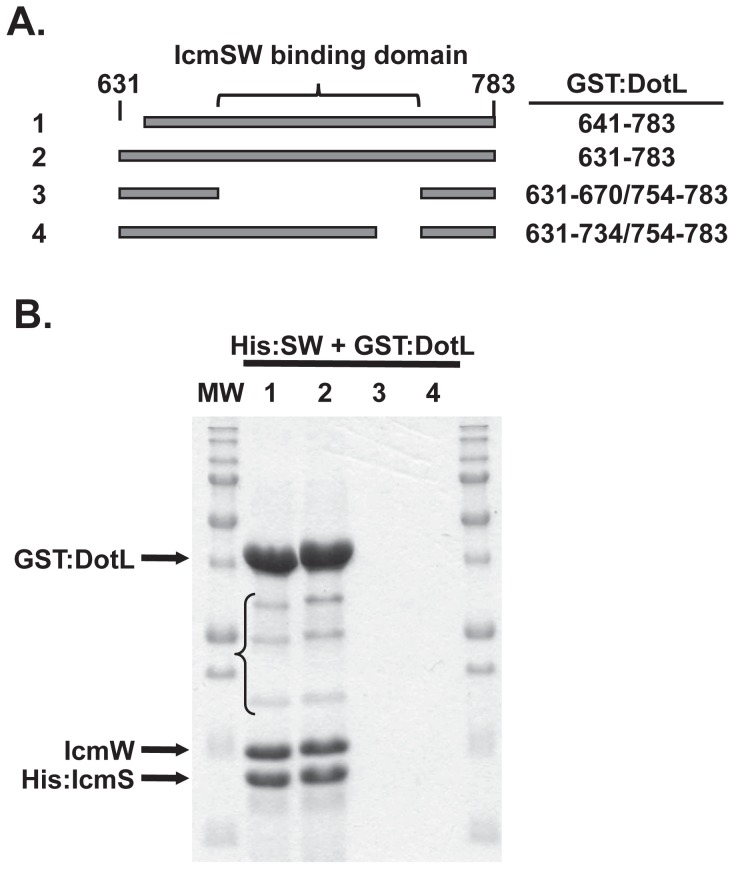
DotL directly interacts with IcmSW. (A) Schematic of four DotL fragments that were assayed for interaction with His:SW. DotL amino acids are indicated on the right and the location of the IcmSW-binding domain is shown at the top. (B) *E. coli* lysates containing His:SW and GST:DotL fragments were sequentially exposed to Ni-NTA and glutathione sepharose resins. Samples were separated on a denaturing SDS-PAGE gel and stained with Coomassie Blue to view isolated proteins. Arrows indicate GST:DotL, IcmW, and His:IcmS proteins, a bracket identify several GST:DotL degradation products, and molecular markers are shown on each side of the gel.

The previous data mapped an IcmSW-binding domain on DotL, and strongly suggested that the connection was direct, but did not rule out the possibility that the interaction was mediated via an intermediate protein that binds both IcmSW and DotL. To eliminate the latter possibility, four different DotL fragments fused to GST were co-expressed with His:SW and subjected to a tandem affinity purification (TAP) procedure [37]. Fragments 1 and 2 include the C-terminal 143 and 153 amino acids of DotL, whereas fragments 3 and 4 contain an internal deletion of 83 and 19 amino acids from fragment 2, thereby removing the IcmSW-binding domain (Fig. 5A). Lysates expressing the DotL fragments and His:SW were sequentially purified on Ni-NTA resin followed by glutathione sepharose resin. The resulting eluent was separated on a SDS-PAGE gel and stained with Coomassie Blue (Fig. 5B).

Three major bands, consisting of GST:DotL, IcmW, and His:IcmS, were recovered when using the DotL fragments 1 and 2 (Fig. 5B). Although three fainter bands were apparent (indicated with a bracket), they appeared to be GST:DotL degradation products based on a modest size shift consistent with the difference in size between fragments 1 and 2 (Fig. 5B) and their detection in an anti-GST western blot (Fig. S7A). Strikingly, no protein was recovered when using fragments 3 or 4, which contain deletions of the IcmSW-binding domain. The failure to detect the DotL fragments from these reactions was likely caused by their inability to bind IcmSW, and not due to their instability or inability to bind the glutathione sepharose resin, since the fragments could be detected and purified if applied directly to the respective resin (Fig. S7B). Thus, tandem purification of GST:DotL and His:SW clearly demonstrates that the interaction between DotL and IcmSW is direct and is not mediated through Dot/Icm substrates, via other T4SS components such as DotM, or a conserved Gram-negative protein.

### The DotL IcmSW-binding site is not required for growth phase dependent destabilization

Although it was clear that DotL could directly bind IcmSW through a domain near its C-terminus, the purpose of this interaction remained enigmatic. A connection between DotL and IcmS was previously observed where DotL became destabilized, due to proteolysis by the ClpAP protease, in a strain lacking *icmS* [18]. Interestingly, degradation of DotL occurred during the transition from exponential phase growth to stationary phase, a point concurrent with the expression of the majority of Dot/Icm substrates [38]. This led to the hypothesis that DotL degradation could be a cellular response of clearing a “jammed” secretion apparatus, which was generated by a failed attempt to export a substrate that was in the wrong conformation due to the absence of IcmSW.

**Figure 6:**
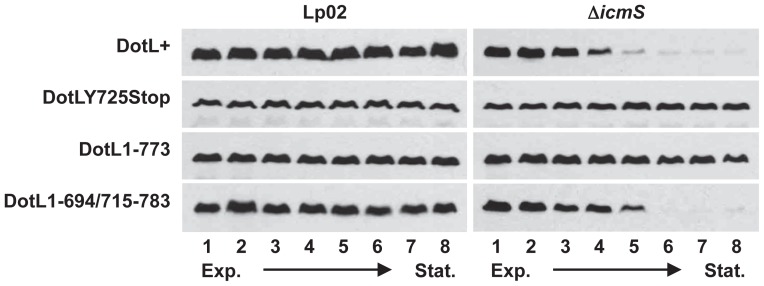
Growth phase dependent instability of DotL in the absence of *icmS*. DotL levels were analyzed by western blotting using a DotL-specific antibody on samples harvested at various stages of growth. Samples include Lp02 or Δ*icmS* strains expressing wild-type DotL, the DotLY725Stop mutant, DotL1–773, or a DotL mutant lacking a domain necessary for IcmSW-binding (DotL1–694/715–783). Samples ranged from mid-exponential phase (lane 1, OD_600_ ∼2.5) to late stationary phase (lane 8, OD_600_ ∼3.5). Results are representative of three independent experiments.

To examine this theory, we compared the steady-state abundance of wild-type DotL with the DotLY725Stop mutant in the presence and absence of IcmS. As previously seen [18], DotL protein levels remain constant in Lp02 but DotL is destabilized in stationary phase in a Δ*icmS* mutant (Fig. 6). Although the amount of the DotLY725Stop protein was unchanged compared to wild-type DotL in the presence of *icmS*, it did not decrease in the absence of the adaptor complex (Fig. 6). One explanation for this result is that IcmSW function as a chaperone to stabilize a poorly folded domain on DotL, and when that domain is removed, DotL levels do not change in the presence or absence of IcmSW. Alternatively, since the *dotL*Y725Stop allele truncates the last 58 amino acids of the DotL protein, it might have simply removed the protease recognition site from DotL. Consistent with latter idea, ClpAP is known to be able to target proteins at either the N- or C-terminus [39].

To differentiate between these two possibilities, we examined if a *dotL* mutant that binds IcmSW, but lacks the last 10 amino acids of DotL, would no longer be degraded in a Δ*icmS* mutant. Similar to the *dotL*Y725Stop mutant, the *dotL*1-773 mutant (originally described in Figure 3B) had unaltered steady-state DotL protein levels in the presence or absence of IcmSW (Fig. 6). This strongly implied that the lack of destabilization of the DotLY725Stop mutant was not due to its inability to bind IcmSW but was due to the absence of a ClpAP recognition site within its C-terminus.

Therefore, to more precisely probe the role of the IcmSW-binding site on DotL protein levels, we employed several of the *dotL* internal deletions that retain DotL's native C-terminus but cannot bind IcmSW (Fig. 4). In contrast to the DotLY725Stop mutant, the DotL1–694/715–783 mutant proteins levels were not affected in the presence of IcmS but exhibited protein instability in the absence of IcmS similar to wild-type DotL protein (Fig. 6). In addition, several other *dotL* mutants that cannot bind IcmSW exhibited a similar phenotype (data not shown). Based on this analysis, it can be concluded that the IcmSW-binding domain on DotL is not involved in modulating DotL's protein levels. Rather, it suggests that DotL destabilization must be due to some other process, perhaps aberrant export of substrates through the Dot/Icm T4SS in the absence of the adaptors.

### The *dotL*Y725Stop mutant is deficient for secretion of only one class of Dot/Icm substrates

Previously we have shown that IcmSW binds directly to DotL, but that this interaction was not required for the assembly of a functional T4SS. However, since the *dotL*Y725Stop mutant has a profound intracellular growth defect, it must be defective for a vital activity such as the translocation of Dot/Icm substrates into host cells. To test if the *dotL*Y725Stop mutant was defective for this process, secretion was assayed in the wild-type strain Lp02, a Δ*icmS* mutant, the *dotL*Y725Stop mutant and a double mutant containing both the *dotL*Y725Stop and the *icmS* deletion. Translocation was measured using reporter fusions consisting of *Bordetella pertussis* adenylate cyclase toxin (CyaA) fused to representative Dot/Icm T4SS effectors, and export was monitored by measuring increased cAMP production by the host cell [40]. In contrast to a previous report [18], secretion was monitored using late exponential cultures to avoid indirect effects due to diminished levels of DotL in the absence of IcmS (see Materials & Methods).

**Figure 7:**
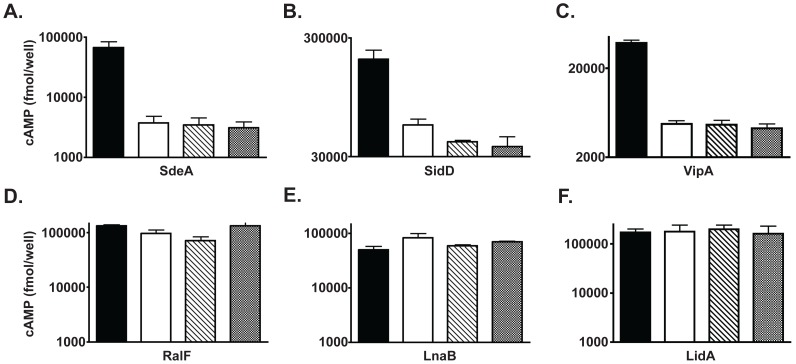
The *dotL*Y725Stop mutant has a specific secretion defect for IcmSW-dependent substrates. Export of CyaA fusions to the following Dot/Icm substrates was assayed: A. SdeA, B. SidD, C. VipA, D. RalF, E. LnaB, and F. LidA. The CyaA:substrate fusions were expressed in a wild-type strain (black bars), a strain lacking *icmS* (white bars), a strain with the *dotL*Y725Stop mutant integrated onto the chromosome (striped bars), and a *dotL*Y725Stop Δ*icmS* double mutant (gray bars). cAMP was measured by ELISA from triplicate wells and error bars represent the standard deviation from the mean.

As previously shown [18], [23], [25], three IcmSW-dependent substrates (SdeA, SidD and VipA) are secreted from a wild-type strain, but exhibit a severe defect in export from a strain lacking *icmS*, confirming their IcmSW-dependence (Fig. 7A–C). Interestingly, secretion of these substrates was severely attenuated in the *dotL*Y725Stop mutant (Fig. 7A–C). Decreased export of the substrates was not due to lowered amounts of IcmSW (data not shown) nor did diminished expression levels of the reporters cause it (Fig. S8A). Moreover, the secretion defect remained in a double mutant consisting of the *dotL*Y725Stop mutant and the *icmS* deletion, indicating that the export deficiency of the *dotL*Y725Stop mutant was not due to an inhibitory effect of expressing IcmSW in the absence of the IcmSW-binding site on DotL.

In contrast to the IcmSW-dependent substrates, three IcmSW-independent substrates (RalF, LnaB, and LidA) [18], [41], [42] were secreted at the same level in all strains, notably including the *dotL*Y725Stop mutant (Fig. 7D–F). A similar result was obtained when export was assayed using a second *dotL* mutant (1–734/754–783 described in Fig. 4B), that contains a smaller, twenty amino acid deletion in the IcmSW-binding site of DotL (Fig. S8B–E). This result confirms that removal of the IcmSW-binding domain on DotL did not prevent the proper assembly or ability to export some substrates from the *L. pneumophila* Dot/Icm T4SS. In addition, the secretion data conclusively demonstrates that the IcmSW-binding site on DotL performs an essential role in the export of IcmSW-dependent substrates, which is independent of the role the adaptors play in directly binding substrates.

## Discussion

In this study, we extensively characterized one *dotL* mutant, *dotL*Y725Stop, which revealed that DotL directly binds to the type IV adaptors IcmSW via a domain that is located near the C-terminus of DotL. Removal of the IcmSW-binding domain from DotL did not prevent the assembly of a functional T4SS, nor did it affect the growth phase instability of DotL that occurs in the absence of IcmS. However, *dotL* mutants unable to bind IcmSW were defective specifically for the export of IcmSW-dependent substrates, thus revealing an additional role for the type IV adaptors in the translocation of a major class of Dot/Icm substrates into the cytoplasm of host cells.

Although the IcmSW complex is critically important for the intracellular replication of *L. pneumophila*, the molecular role of these two proteins has remained elusive. Based on the type IV adaptors resemblance to type III secretion chaperones, including their small size, acidic nature, and ability to dimerize, it was initially proposed that IcmS and IcmW might function as chaperones to mediate the export of Dot/Icm substrates [22], [24]. It was subsequently shown that secretion of several substrates, including SdeA, SidG, SidH, and WipA, was more dependent on IcmSW than the substrate RalF, whose export was largely IcmSW-independent [23], [24]. Moreover, consistent with their proposed role as secretion chaperones, several of the substrates that require IcmSW for their optimal export were also shown to bind IcmSW [22]–[25]. Conversely, RalF does not require IcmSW for its export and was unable to bind to IcmSW [22]–[25]. To date, 24 out of 34 Dot/Icm substrates examined require IcmS and/or IcmW for their export (summarized in Table S2). However, it is worth noting that this collection includes only a small fraction of the ∼300 known Dot/Icm substrates [5]. In addition, only six substrates have been assayed for their ability to bind the type IV adaptors (Table S2).

Although a good correlation exists between these traits within this limited data set, it is not known how the adaptors mediate export by binding to IcmSW-dependent substrates. Direct binding of substrates by the type IV adaptors is reminiscent of one or more of the proposed roles performed by type III secretion chaperones [43], [44]. For example, IcmSW-binding of substrates could potentially stabilize or prevent aggregation of the substrates in the bacterial cytoplasm. Though, this does not appear to be a general task for the *L. pneumophila* type IV adaptors as SidE family members and SidG are stable and do not aggregate in the absence of IcmSW [23], [25].

Alternatively, IcmSW could function to target substrates to the T4SS apparatus. However, IcmSW binds SidG at a location distinct from its C-terminal signal sequence and removal of the IcmSW-binding domain(s) allows SidG to be exported in type IV adaptor-independent manner, inconsistent with IcmSW functioning directly as a targeting factor [25]. Instead Cambronne *et al*. proposed that the type IV adaptors might regulate secretion by preventing occlusion of the C-terminal signal sequence in a partially folded domain of the substrate [25]. While this is a novel and interesting concept, no experimental evidence exists that IcmSW-dependent substrates fail to engage the T4SS apparatus in the absence of the type IV adaptors. In contrast, our data on DotL protein levels suggests that IcmSW-dependent substrates can interact with DotL in the absence of *icmS*, leading to the degradation of DotL.

Therefore, IcmSW likely perform an alternative role in mediating export of many Dot/Icm substrates. Since IcmSW appear not to be required for targeting, and are not exported along with the Dot/Icm substrates, they likely act at the membrane complex perhaps by directly assisting in the export of the proteins. Consistent with this notion, we have now discovered that IcmSW bind directly to DotL and this interaction is critical for the export of IcmSW-dependent substrates. Precedence exists for an interaction between T3SS chaperones and their associated ATPases during targeting of substrates to the secretion apparatus. In addition, some T3SS chaperones are able to interact with their corresponding ATPase in the absence of their cognate substrate, although it is not clear why they interact nor has the interaction been shown to be required for secretion (reviewed in [43], [45]).

**Figure 8:**
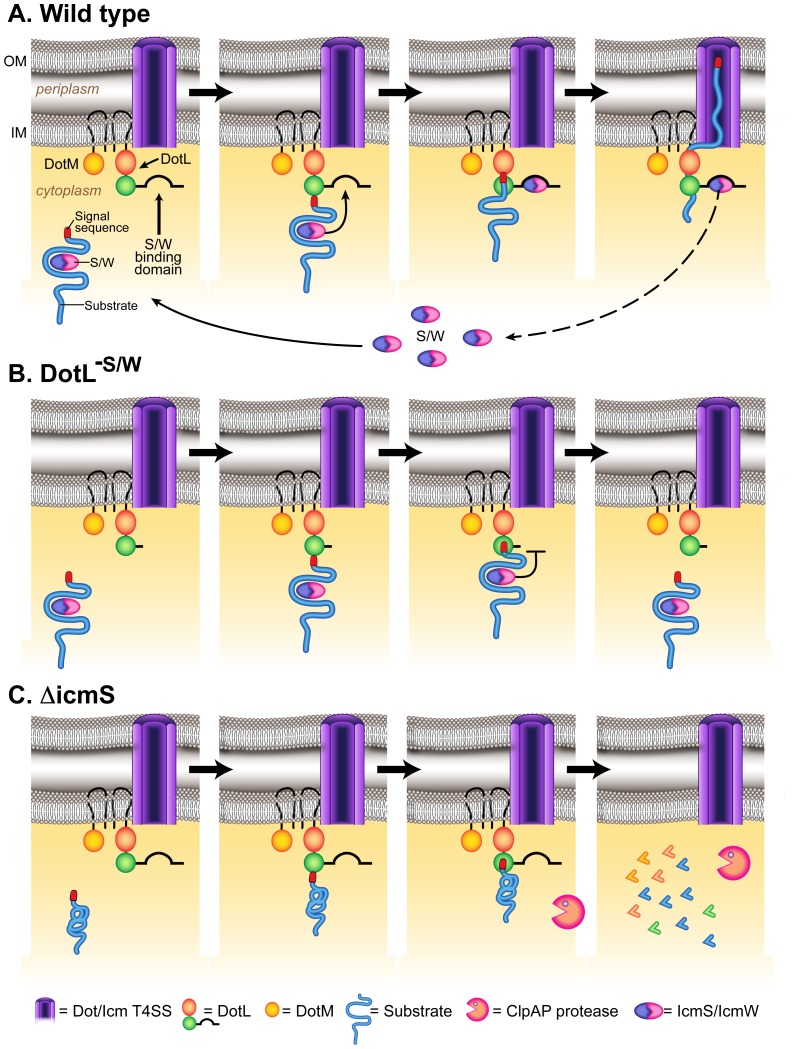
Model for secretion of IcmS/IcmW-dependent effectors by DotL. The transmembrane-spanning *L. pneumophila* T4SS complex is represented by a purple cylinder, components of the T4CP subcomplex (DotL, DotM, IcmS, and IcmW) are labeled and the inner and outer membranes are indicated as IM and OM, respectively. (A) Export of IcmSW-dependent substrates in a wild type strain. (B) Rejection of IcmSW-dependent substrates in a *dotL* mutant strain that lacks the IcmSW-dependent binding domain (*dotL* ^−S/W^). (C) Jamming of IcmSW-dependent substrates in a Δ*icmS* mutant, leads to DotL degradation by ClpAP.

Therefore, taking all of the existing data into context, we propose a new model for the export of IcmSW-dependent substrates by the *L. pneumophila* Dot/Icm T4SS (Fig. 8A). In this unified model, IcmSW-dependent substrates are bound by the type IV adaptors in the cytoplasm in order to maintain the substrates in a translocation competent form. Substrates are targeted to the inner membrane via their C-terminal signal sequence and likely interact directly with the type IV coupling protein DotL. After the substrates engage the translocation apparatus, IcmSW need to be removed from the substrates and this may be mediated by transfer to the IcmSW-binding domain on DotL, perhaps while the substrates are being actively pumped out of the cell. Later, IcmSW would dissociate from DotL, disengage from the inner membrane, and transit back to the cytoplasm in order to interact with new substrates, thus completing an export cycle (Fig. 8A). Although this model incorporates all of the existing data, and provides a reasonable explanation for how IcmSW could function while independently binding substrates and DotL, there are a number of potential caveats. For example, the model predicts a large amount of IcmSW would be required in the cell in order to bind the vast number of type IV-adaptor dependent substrates. This has not been experimentally established nor has the direct transfer of IcmSW from substrates to DotL.

Nevertheless, our working model allows us to speculate why IcmSW-dependent substrates are not exported in different mutant backgrounds that are compromised for type IV adaptor activity. For example, a strain that expresses a *dotL* mutant that is unable to bind IcmSW (*dotL* ^−S/W^) is not capable of exporting IcmSW-dependent substrates (Fig. 8B). In this case, IcmSW-dependent substrates would still be bound by the adaptors and maintained in a translocation competent form. Since targeting is dependent on their C-terminal signal sequence, these substrates would still bind to inner membrane components of the T4SS. However, as the substrates engage the T4SS apparatus, the proposed transfer of IcmSW from the substrate onto DotL would fail because no acceptor site would be present. As a result, the substrate might be rejected from the apparatus, a result previously observed with T3SS substrates that are fused to proteins that are difficult to be unfolded such as DHFR and ubiquitin (reviewed in [46]). This model of rejection is consistent with our observation that DotL^−S/W^ is not degraded in wild-type strains expressing IcmS.

In contrast, the phenotypes associated with a Δ*icmS* mutant are more complicated because both IcmSW-dependent substrates and DotL would not bind the type IV adaptors (Fig. 8C). Although the *dotL* ^−S/W^ mutant and a Δ*icmS* mutant are each unable to export IcmSW-dependent substrates, we propose that their molecular defects differ. In the case of a strain lacking *icmS*, IcmSW-dependent substrates would still interact with DotL via their C-terminal signal sequence, however they would not be in a translocation competent form due to the absence of the type IV adaptors. As a result, the substrates might engage the apparatus in an aberrant manner and become “jammed”. This would likely induce membrane stress resulting in removal of the DotL/substrate aggregate via the ClpAP protease, which would also be consistent with our results on DotL protein levels in the absence of *icmS*. Furthermore, this model predicts that substrates can interact with DotL in the absence of the type IV adaptors, thus supporting the idea that IcmSW do not perform a role in targeting of substrates to the Dot/Icm apparatus.

By characterizing the *dotL*Y725Stop mutant, we discovered that DotL directly binds to the type IV adaptors and that the interaction is required for the specific export of IcmSW-dependent substrates. This mutant provides a key reagent for the future dissection of the role of IcmSW, as the phenotypes of the Δ*icmS* mutant are clearly pleiotropic. Moreover, as IcmSW-dependent substrates appear to constitute the major class of Dot/Icm proteins, understanding how *L. pneumophila* controls and regulates the secretion of several hundred proteins will be vital to understanding how this pathogen is able to replicate inside host cells and cause disease.

## Materials and Methods

### Ethics statement

This study was carried out in strict accordance with the recommendations in the Guide for the Care and Use of Laboratory Animals of the National Institutes of Health. The protocol was approved by the Institutional Animal Care and Use Committee at the Washington University School of Medicine (Assurance Number: A3381-01). All efforts were made to minimize suffering.

### Bacterial strains and media

Strains used in this study are provided in Table S3. All *Legionella pneumophila* strains having a JV number are derived from the wild type derivative, *L. pneumophila* Philadelphia Lp02 (*hsdR rpsL thyA*), of the clinical isolate *L. pneumophila* Philadelphia-1 [47]. *L. pneumophila* strains were grown in yeast extract broth (AYE) or on solid media consisting of charcoal yeast agar (CYE) both buffered with *N*-(2-acetamido)-2-aminoethanesulfonic acid (ACES) [48]. Antibiotics, sucrose (5%), and thymidine (100 µg/ml) were added as needed.

### Screen for *dotL* mutants

Twelve *dotL* mutant libraries were generated by PCR using Taq DNA polymerase, the primers JVP1038 (CCCGAATTCGGAATTAGAGCCATGATGCG) and JVP1039 (CCCGCATGCCACTTCTACCTCCAATTGCCG) and a *dotL* complementing clone (pJB3151) as the template. The PCR fragment was cloned into an IPTG inducible vector (pJB4858), transformants were pooled into libraries, and plasmid DNA was isolated. Mutant libraries were transformed into a *dotL*/Δ*dotL*::Cm^R^ merodiploid strain (JV1003). Integrants were pooled and plated on CYE plates containing 5% sucrose, 0.1 mM IPTG, 2 µg/ml chloramphenicol to select for dotL plasmids that allowed resolution of the merodiploid to Δ*dotL*::Cm^R^. The putative *dotL* mutants were used to infect *Acanthamoeba castellanii* and a visual assessment for growth was performed at 24 and 48 hours. *dotL* mutants that did not replicate in *A. castellanii* were recovered and confirmed for plasmid linkage. Approximately 100 *dotL* mutant plasmids that had an attenuated growth phenotype were sequenced at the Harvard Biopolymer Facility to identify amino acid changes responsible for the growth defect. Twelve clones contained one amino acid change and the remaining contained two or more amino acid changes; only mutants with a single amino acid change were pursued.

### Complementation of Δ*dotL* lethality by the *dotL* mutants

*dotL* mutants were transformed into the *dotL* merodiploid strain, Lp02 *dotL*/Δ*dotL*::Cm^R^ (JV1003), and an OD600 of 1.0 suspension from a two day patch was diluted and plated on CYE sucrose IPTG chloramphenicol plates to select for resolution to Δ*dotL*::Cm^R^ and on CYE + IPTG to determine the total number of cells. The frequency of resolution to Δ*dotL* was determined by dividing the number of cells that resolved to Δ*dotL*::Cm^R^ by the total number of cells.

### Growth of *L. pneumophila* in host cells

*L. pneumophila* liquid cultures were grown with appropriate selection to the point of infectivity as determined by OD600 and motility. Bacteria were washed, resuspended at an OD600 of 1.0 and diluted 1∶10,000 for infection. *A. castellanii* were infected, lysed with 5% saponin in phosphate buffered saline (PBS) at 0, 20, 31, and 42 hours, and dilutions were plated on CYE + IPTG to determine rates of multiplication. Primary bone marrow derived macrophages were prepared as previously described [31] and infected as described with the *A. castellanii*. Macrophages were lysed at 24 hour intervals with water and fold growth was determined by plating dilutions on CYE + IPTG.

### Immunoblot analysis

Samples were collected, resuspended in Laemmli sample buffer, boiled for 5 minutes, separated by SDS-PAGE gel electrophoresis and transferred to PVDF membranes. Membrane was blocked with PBS containing 5% non-fat dry milk, washed with 0.05% Tween 20 in PBS, and incubated for 1 hour with primary antibody diluted in the non-fat dry milk solution. Blots were then washed and incubated for 1 hour with secondary goat anti-rabbit or goat anti-mouse conjugated to horseradish peroxidase (Sigma) diluted in non-fat dry milk solution. Finally, blots were washed and developed using an ECL detection kit (Amersham Biosciences).

### Determination of cellular location by membrane fractionation

Membrane fractionation was performed as previously described [17]. Briefly, *L. pneumophila* liquid cultures were grown to the point of infectivity as above and 20 OD600 of cells were collected. Cell pellets was resuspended in lysis buffer (50 mM Tris pH 8.0, 0.2 mg/ml lysozyme, and protease inhibitor cocktail (Sigma) and lysed by French press (14,000 PSI). Unlysed cells were removed by centrifugation at 10,000 *g* for 10 minutes. Cytoplasmic and membrane proteins were distinguished by ultracentrifugation at 100,000 *g* for 1 hour. Membrane proteins were resuspended in 1× Laemmli sample buffer and all other fractions were diluted with 2× Laemmli sample buffer. Fractionations were analyzed by immunoblot analysis as above. Fractionation quality was assessed by immunoblot analysis with antibodies to the cytoplasmic protein isocitrate dehydrogenase (ICDH) and the inner-membrane protein LepB.

### Interaction of GST:DotL fragments with His:SW

His and GST fusion plasmids were co-expressed in *E. coli*, induced with IPTG for four hours at room temperature, and 10 OD600 of cells were collected. Cell pellets were resuspended in lysis buffer (50 mM Tris-pH 8, 150 mM NaCl, 1% TritonX-100, 1 mM DTT, 1 mM PMSF, 10 mM imidazole) and lysed by sonication. Unlysed cells were removed by centrifugation at 10,000 *g* for 20 minutes. Cell lysates were added to a 50 µl Ni-NTA (Qiagen) bed volume and incubated with rotation for 2 hours at 4 C. Ni-NTA was washed twice in wash buffer (lysis buffer with 30 mM imidazole) and proteins were eluted in 2× Laemmli sample buffer by boiling for 5 minutes. Fractions were loaded proportionally and analyzed by immunoblot analysis as above with specific His and GST antibodies.

### Contact dependent cytotoxicity

Contact dependent cytotoxicity was performed as previously described [33], [49]. Briefly, *L. pneumophila* cultures were grown to the point of infectivity as described above, washed, and pelleted onto BMM by centrifugation at a multiplicity of infection (MOIs) of 5, 50, or 500. BMM were infected for one hour, washed, stained with a 5∶1 mixture of ethidium bromide and acridine orange to assess cell viability and immediately examined by immunofluorescent microscopy. The ability to induce contact-dependent cytotoxicity is expressed as the percentage of ethidium bromide positive cells and calculated by dividing the total number of ethidium bromide positive cells by the total number of cells in three randomly selected fields.

### Plasmid transfer assay

Plasmid transfer assays were performed as previously described [13]. Briefly, *L. pneumophila* strains were grown in liquid media to the point of infectivity as described above. *L. pneumophila* strains were mixed with the *E. coli* recipient ER1821 in a 10∶1 ratio on a 0.45 µm filter (Millipore) on non-selective CYET media for two hours. Bacteria were washed from the mating membrane with sterile water and assayed for the number of plasmid transfer events by plating on selective media. Rate of plasmid transfer was determined by dividing the number of *E. coli* recipients by the total number of *L. pneumophila* donors.

### DotL protein levels

*L. pneumophila* strains were grown in liquid media. When cultures reached early exponential phase growth rate was monitored by OD600 and 1 OD600 of cells were collected every hour for fifteen hours. Protein samples were prepared as above and assayed by western blot with a specific DotL antibody to determine protein levels.

### Adenylate cyclase reporter assays for substrate secretion

Reporter assays were performed as described [18], [23] with the following modification. In Vincent et al, it was shown that DotL becomes destabilized in stationary phase in the absence of IcmS resulting in diminished export of IcmSW-independent substrates such as RalF and this defect could be suppressed by over-producing DotL. This indirect defect could be avoided by assaying export of substrates in late exponential phase, prior to the point where DotL levels decrease. Measuring secretion of substrates at either point in growth is feasible since the Dot/Icm system is constitutively expressed and is functional at all phases of growth.

Specifically, CyaA fusions were transformed into the *L. pneumophila* strains Lp02, Lp02 Δ*icmS* (JV1962), Lp02 *dotL*Y725Stop (JV7325) and Lp02 *dotL*Y725Stop Δ*icmS* (JV7645). The strains were grown in liquid media and induced for 2.5 hours with IPTG at early exponential phase. 1 OD600 of cells was collected, washed, diluted 1∶200 in RPMI-1640 (HyClone) supplemented with fetal bovine serum 10% (FBS) (HyClone) for infection of U937 cells. Bacterial cultures were pelleted onto differentiated U937 cells for 5 minutes at 1000 *g* at room temperature and infections were allowed to proceed for 1 hour at 37 C. Cells were washed 3× in warm PBS, lysed (50 mM HCl, 0.1% Triton X-100) and immediately boiled for 5 minutes. Subsequently HCl was neutralized with NaOH and cAMP was extracted was extracted with 95% ethanol. Extracted cAMP was desiccated and analyzed via a competitive ELISA (cAMP Biotrak Enzymeimmunoassay System, Amersham or Cyclic AMP EIA Kit, Cayman Chemical).

## Supporting Information

Figure S1 **The** ***dotL*** **mutants complement Δ** ***dotL*** **lethality and express stable protein.** (A) A *dotL* merodiploid strain (*dotL*/Δ*dotL*::Cm^R^) containing a *dotL* complementing plasmid, vector, or the *dotL* mutants was monitored for its ability to resolve to the Δ*dotL*::Cm^R^ locus. The experiment was performed in triplicate and error bars represent the standard deviation from the mean. (B) Westerns using antibodies specific for DotL or DotM were performed on each of the *dotL* mutants. Westerns to the cytoplasmic housekeeping protein ICDH serve as a loading control. Results are representative of three independent experiments.
(TIF)

Figure S2 **The** ***dotL*** **mutant's exhibit an intracellular growth defect inside host cells.** *L. pneumophila* was used to infect *A. castellanii* (A–B) or BMMs (C–D). Growth is shown for a wild-type strain (Lp02) (filled squares), a T4SS-deficient strain (Lp03) (filled triangles), and a Δ*dotL* strain containing a *dotL* complementing clone (filled inverted triangles) or the *dotL* mutants (open symbols). Error bars represent the standard deviation from the mean and the results are representative of three independent experiments.
(TIF)

Figure S3 **Controls for** ***Legionella*** **fractionations showing IcmSW recruitment to the membrane.** *L. pneumophila* cultures were lysed and fractionated as described in the Materials & Methods. Protein fractions were analyzed by western blotting with the specified antibody to ensure the quality of the separation technique. (A) Westerns detecting the cytoplasmic housekeeping protein isocitrate dehydrogenase (ICDH) demonstrate that no cytoplasmic proteins are present in the membrane fractions. (B) Westerns detecting the inner membrane signal peptidase, LepB, demonstrate that membrane proteins are not present in the cytoplasmic fractions. The control in lane 1 is a total protein fraction from the wild-type strain loaded in the T, S, and M blots to illustrate protein transfer to the membrane occurred.
(TIF)

Figure S4 ***dotL*** **Y725Stop exhibits contact-dependent cytotoxicity.** BMMs were challenged with four different *L. pneumophila* cultures (wild-type strain Lp02, the *dotA* mutant Lp03, a Δ*dotL*::Cm^R^ strain containing a wild-type *dotL* ^+^ complementing clone or the *dotL*Y725Stop clone). Infections were done using various amounts of bacteria including: uninfected (light gray bar), MOI of 5 (black bar), MOI of 50 (white bar), and MOI of 100 (gray bar). Host cell permeabilization was assayed using a live/dead stain consisting of acridine orange and ethidium bromide (EtBr). Results are displayed as the percentage of EtBr-positive macrophages and were determined by taking the number of EtBr positive cells divided by the total number of host cells in three random fields. The results are representative of several independent experiments and the standard deviation from the mean is shown for each sample.
(TIF)

Figure S5 **Controls for delineation of the IcmSW-binding domain of DotL.** (A) GST:DotL fragments that interacted with His:SW in Fig. 3A were tested to confirm that the interaction was specific. GST:DotL fragments were co-expressed with empty His fusion vector assayed for non-specific binding to Ni-NTA resin. Protein from the total (T), flow-through (F), and bound fractions (B) were assessed by western blotting with a GST-specific antibody. (B) Samples from Fig. 3A were blotted with an anti-His antibody to demonstrate near complete retention of His:SW to the Ni-NTA resin. (C) Selected samples from Fig. 3B were examined to confirm the quality of the membrane fractionation technique. As described in Fig. S4, ICDH is a cytoplasmic protein and LepB is an inner membrane protease. (D) Samples from Figures 3B, 3C, 4A and 4B were analyzed by western blotting with DotL-specific antibodies to assay the total amount of DotL protein in the cells. ICDH blots served as a loading control.
(TIF)

Figure S6 **Controls for identifying the IcmSW-domain that is sufficient to bind DotL.** (A) Longer fragments of GST-DotL starting at amino acid 671 bind to IcmSW but shorter fragments become unstable. GST-DotL was detected by western blots using a GST-specific antibody. (B) GST-DotL fragments from Fig. 4C were tested to confirm that the interaction was specific as described in Fig. S5A. (C) Samples from Fig. 4C were blotted with an anti-His antibody to demonstrate near complete retention of His:SW to the Ni-NTA resin.
(TIF)

Figure S7 **DotL and IcmSW directly interact.** (A) Non-stoichiometric bands from the tandem affinity purification are GST:DotL degradation products. Samples used in Fig. 5 were separated on an SDS-PAGE gel, probed with a GST specific antibody, revealing several degradation products. Fragment 2 was also loaded on the right side of the gel as a control. (B–D) Lysates containing His:SW and the four fragments from Fig. 5 were purified on either a Ni-NTA column (Ni) or a glutathione sepharose column (G). Elutions were run on SDS-PAGE gel and stained with Coomassie (B), probed with anti-His antibody to detect His-IcmS (C) or probed with anti-IcmW antibody (D). His:SW is able to bind Ni-NTA in all cases and all 4 GST:DotL fragments are able to bind glutathione sepharose. GST:DotL fragments 1 & 2 co-purify from the Ni column, but fragments 3 & 4 do not. His:SW co-purifies from the glutathione sepharose column when using fragments 1 & 2 but not with fragments 3 & 4. The * indicates a His:SW dimer that was not denatured by the SDS-PAGE gel and can be detected in the westerns (C & D).
(TIF)

Figure S8 **The DotL1–734/754–783 mutant is defective for secretion of only IcmSW-dependent substrates as observed for the original DotLY725Stop mutant.** (A) Protein levels of CyaA fusions to Dot/Icm substrates used in Figure 7 were assessed by western blot with a CyaA-specific antibody and shown to produce equivalent levels of protein. (B & C) Export of CyaA fusions to IcmSW-dependent effectors (SdeA, SidD, VipA) and IcmSW-independent effectors (RalF, LnaB, LidA) were assayed in a wild-type strain (black bars), Δ*icmS* mutant (white bars), and a strain with DotL1–734/754–783 integrated onto the chromosome (striped bars). cAMP was measured from triplicate wells and error bars represent the standard deviation from the mean. (D & E) Protein samples from Fig. S8B and S8C were analyzed as in Fig. S8A.
(TIF)

Table S1 ***dotL*** **Y725Stop is able to transfer an RSF1010 mobilizable plasmid.** Mobilization of the RSF1010 plasmid by the *L. pneumophila* T4SS to *E. coli* recipients was determined. The rate of transfer was calculated by dividing the average number of recipients by the average total number of donor cells.
(PDF)

Table S2 **IcmSW dependence of Dot/Icm substrates.**
(PDF)

Table S3 **Relevant strains, plasmids, and primers employed in this study.**
(PDF)

Table S4 **Construction of plasmids employed in this study.**
(PDF)
