# Supplementary figures and images for: The Legionella IcmSW Complex Directly Interacts with DotL to Mediate Translocation of Adaptor-Dependent Substrates
Source: PLoS Pathog. 2012 Sep 13;8(9):e1002910. doi: 10.1371/journal.ppat.1002910 (PMC3441705; doi:10.1371/journal.ppat.1002910)

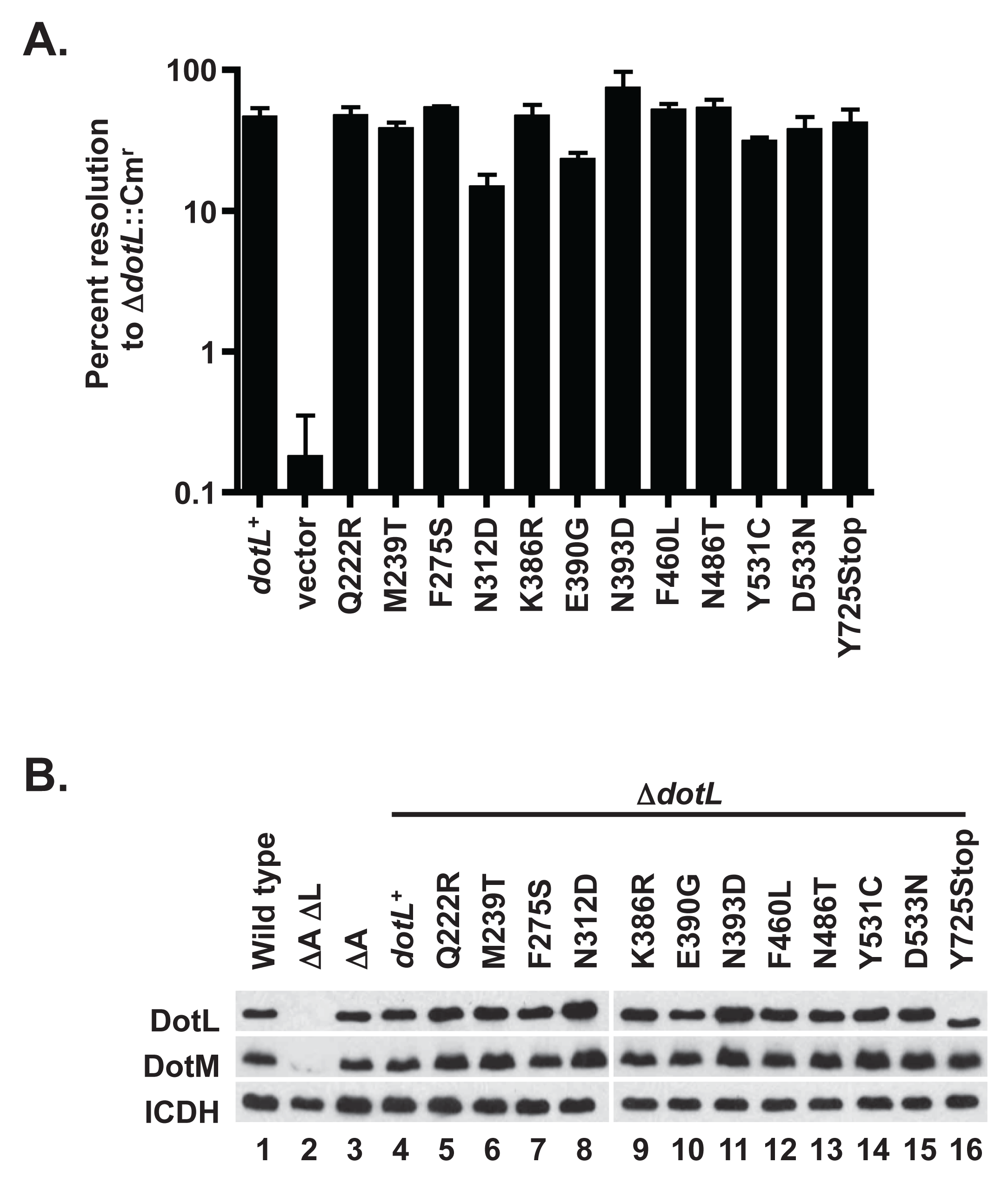

Supplement: Figure S1 — The dotL mutants complement Δ dotL lethality and express stable protein. (A) A dotL merodiploid strain (dotL/ΔdotL::CmR) containing a dotL complementing plasmid, vector, or the dotL mutants was monitored for its ability to resolve to the ΔdotL::CmR locus. The experiment was performed in triplicate and error bars represent the standard deviation from the mean. (B) Westerns using antibodies specific for DotL or DotM were performed on each of the dotL mutants. Westerns to the cytoplasmic housekeeping protein ICDH serve as a loading control. Results are representative of three independent experiments. (TIF) [file ppat.1002910.s001.tif]

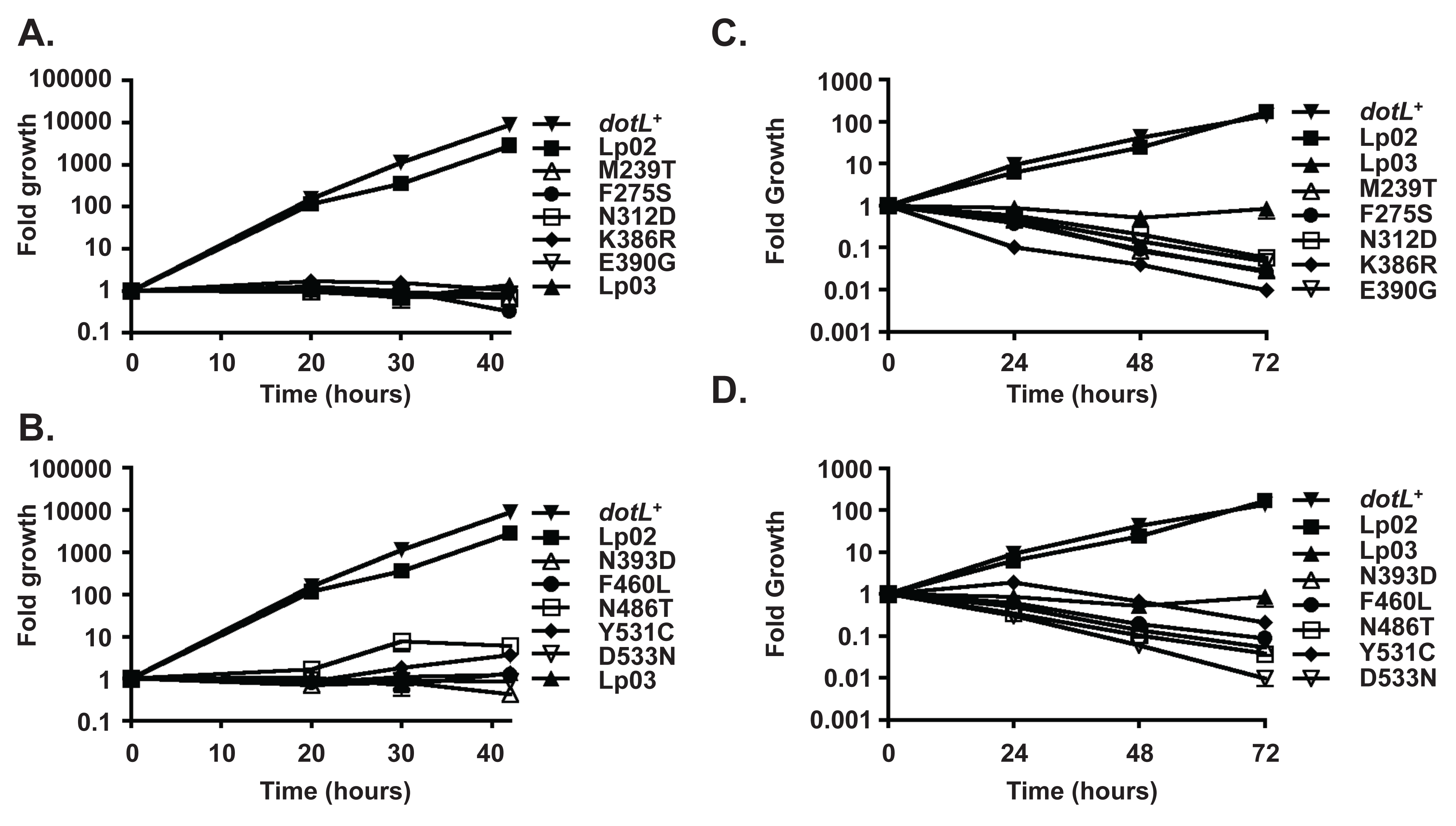

Supplement: Figure S2 — The dotL mutant's exhibit an intracellular growth defect inside host cells. L. pneumophila was used to infect A. castellanii (A–B) or BMMs (C–D). Growth is shown for a wild-type strain (Lp02) (filled squares), a T4SS-deficient strain (Lp03) (filled triangles), and a ΔdotL strain containing a dotL complementing clone (filled inverted triangles) or the dotL mutants (open symbols). Error bars represent the standard deviation from the mean and the results are representative of three independent experiments. (TIF) [file ppat.1002910.s002.tif]

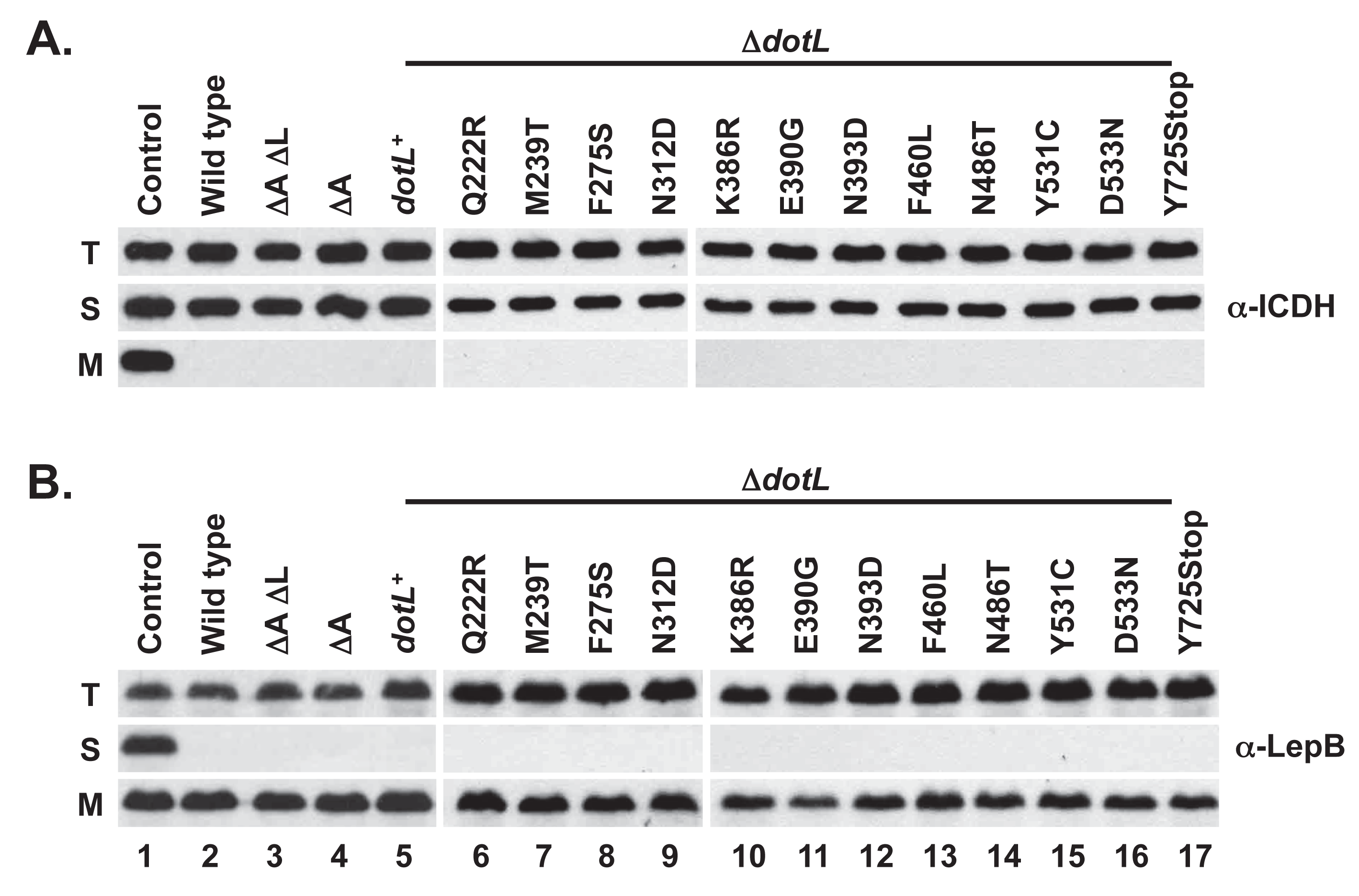

Supplement: Figure S3 — Controls for Legionella fractionations showing IcmSW recruitment to the membrane. L. pneumophila cultures were lysed and fractionated as described in the Materials & Methods. Protein fractions were analyzed by western blotting with the specified antibody to ensure the quality of the separation technique. (A) Westerns detecting the cytoplasmic housekeeping protein isocitrate dehydrogenase (ICDH) demonstrate that no cytoplasmic proteins are present in the membrane fractions. (B) Westerns detecting the inner membrane signal peptidase, LepB, demonstrate that membrane proteins are not present in the cytoplasmic fractions. The control in lane 1 is a total protein fraction from the wild-type strain loaded in the T, S, and M blots to illustrate protein transfer to the membrane occurred. (TIF) [file ppat.1002910.s003.tif]

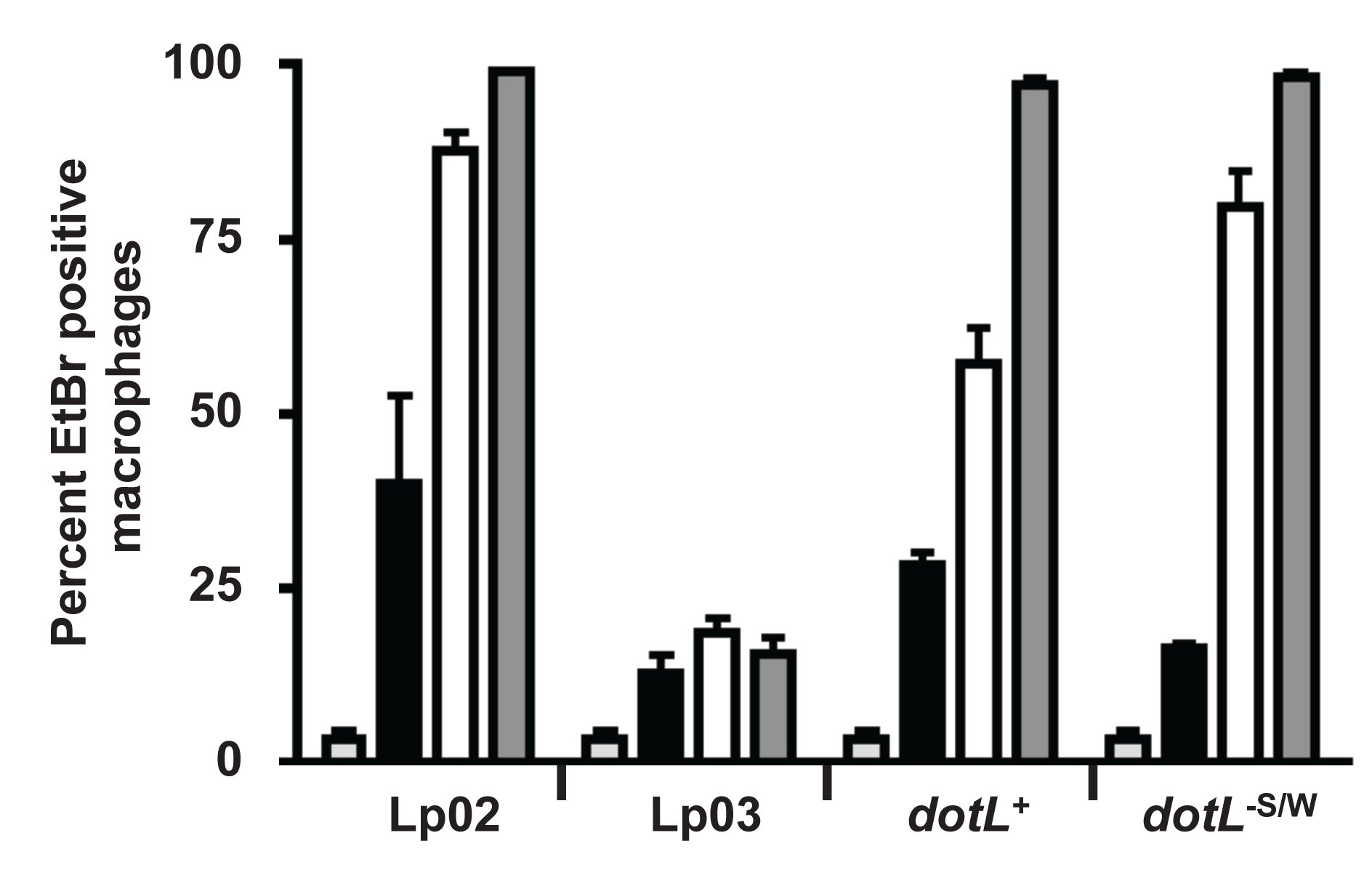

Supplement: Figure S4 — dotL Y725Stop exhibits contact-dependent cytotoxicity. BMMs were challenged with four different L. pneumophila cultures (wild-type strain Lp02, the dotA mutant Lp03, a ΔdotL::CmR strain containing a wild-type dotL + complementing clone or the dotLY725Stop clone). Infections were done using various amounts of bacteria including: uninfected (light gray bar), MOI of 5 (black bar), MOI of 50 (white bar), and MOI of 100 (gray bar). Host cell permeabilization was assayed using a live/dead stain consisting of acridine orange and ethidium bromide (EtBr). Results are displayed as the percentage of EtBr-positive macrophages and were determined by taking the number of EtBr positive cells divided by the total number of host cells in three random fields. The results are representative of several independent experiments and the standard deviation from the mean is shown for each sample. (TIF) [file ppat.1002910.s004.tif]

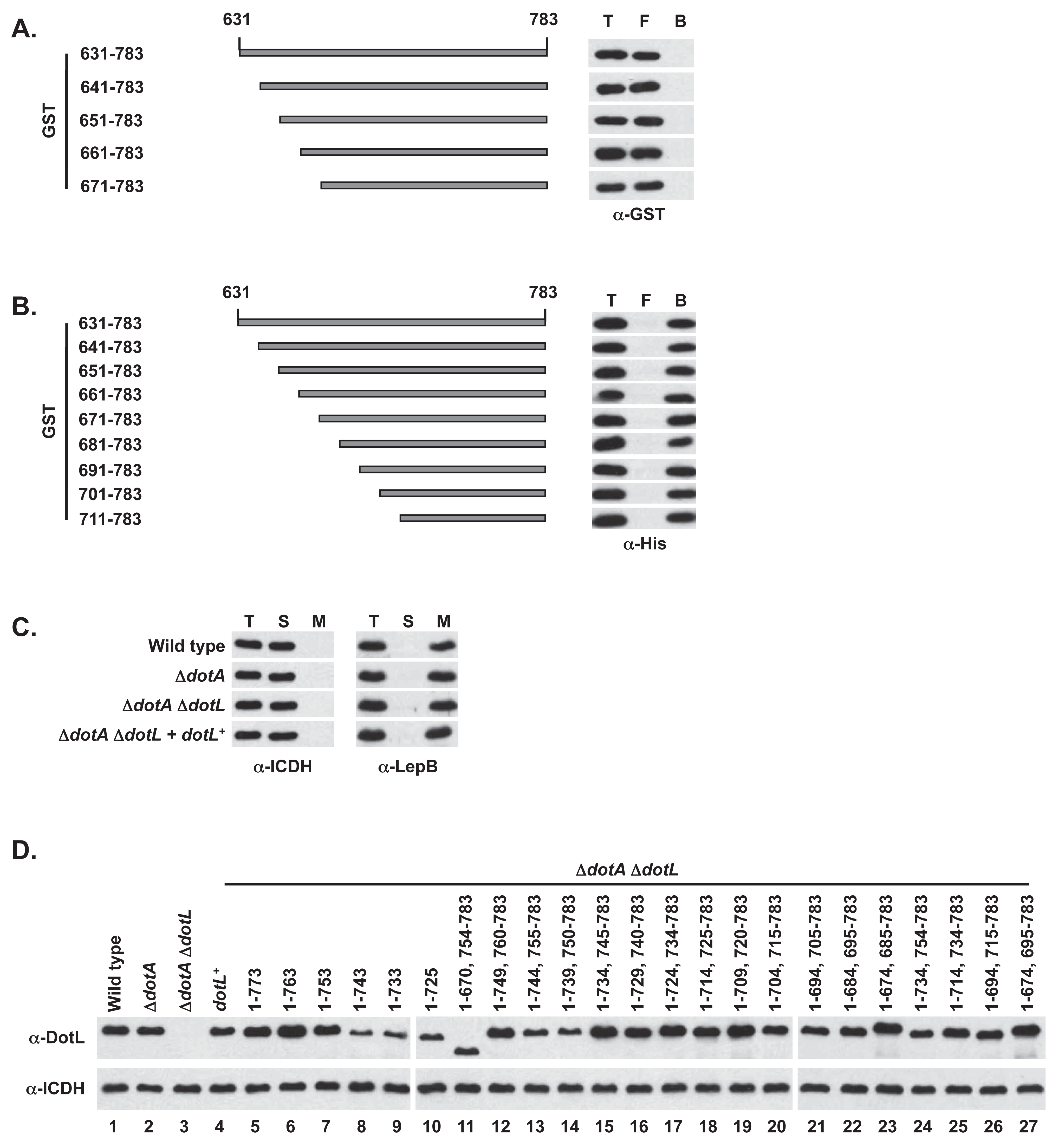

Supplement: Figure S5 — Controls for delineation of the IcmSW-binding domain of DotL. (A) GST:DotL fragments that interacted with His:SW in Fig. 3A were tested to confirm that the interaction was specific. GST:DotL fragments were co-expressed with empty His fusion vector assayed for non-specific binding to Ni-NTA resin. Protein from the total (T), flow-through (F), and bound fractions (B) were assessed by western blotting with a GST-specific antibody. (B) Samples from Fig. 3A were blotted with an anti-His antibody to demonstrate near complete retention of His:SW to the Ni-NTA resin. (C) Selected samples from Fig. 3B were examined to confirm the quality of the membrane fractionation technique. As described in Fig. S4, ICDH is a cytoplasmic protein and LepB is an inner membrane protease. (D) Samples from Figures 3B, 3C, 4A and 4B were analyzed by western blotting with DotL-specific antibodies to assay the total amount of DotL protein in the cells. ICDH blots served as a loading control. (TIF) [file ppat.1002910.s005.tif]

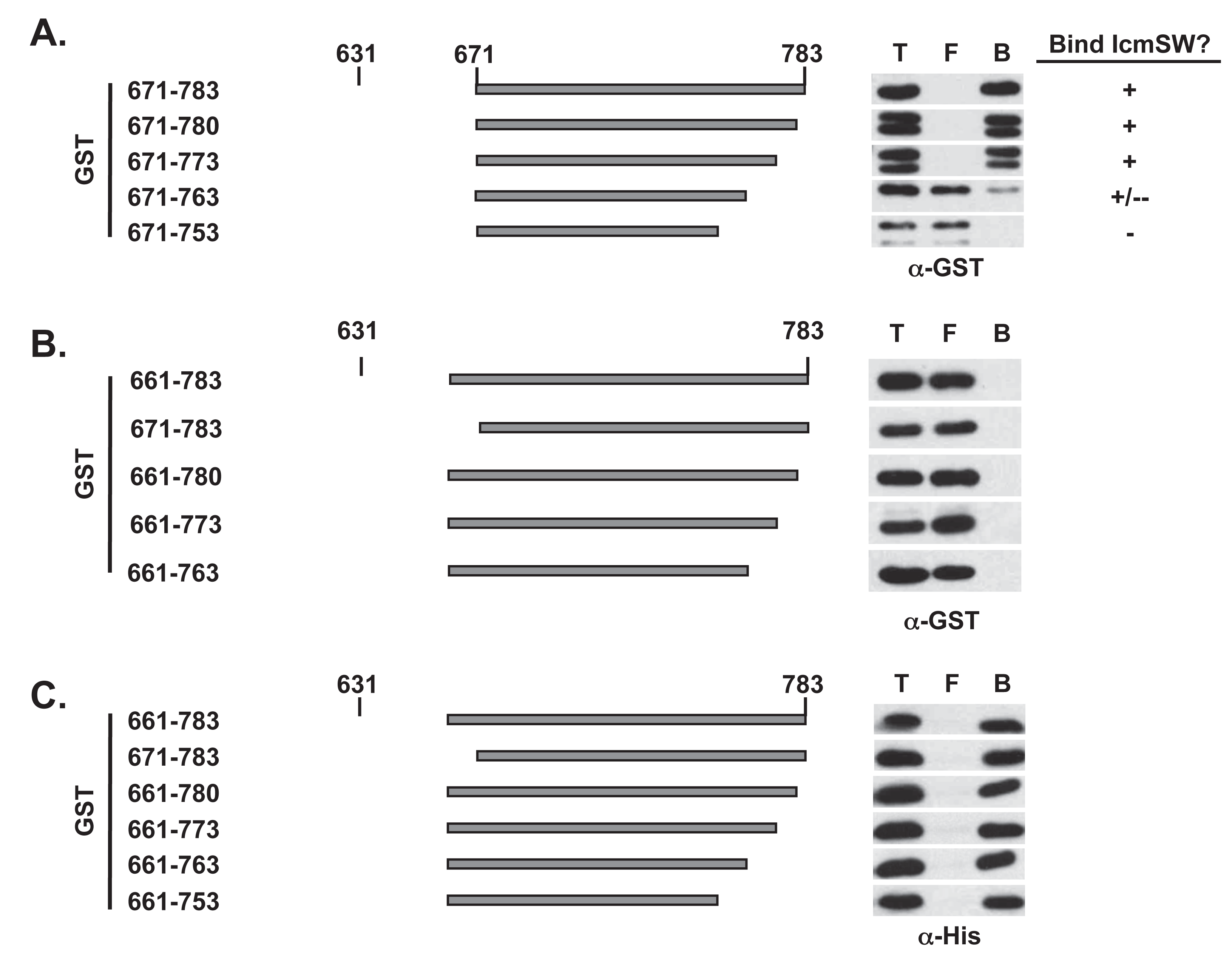

Supplement: Figure S6 — Controls for identifying the IcmSW-domain that is sufficient to bind DotL. (A) Longer fragments of GST-DotL starting at amino acid 671 bind to IcmSW but shorter fragments become unstable. GST-DotL was detected by western blots using a GST-specific antibody. (B) GST-DotL fragments from Fig. 4C were tested to confirm that the interaction was specific as described in Fig. S5A. (C) Samples from Fig. 4C were blotted with an anti-His antibody to demonstrate near complete retention of His:SW to the Ni-NTA resin. (TIF) [file ppat.1002910.s006.tif]

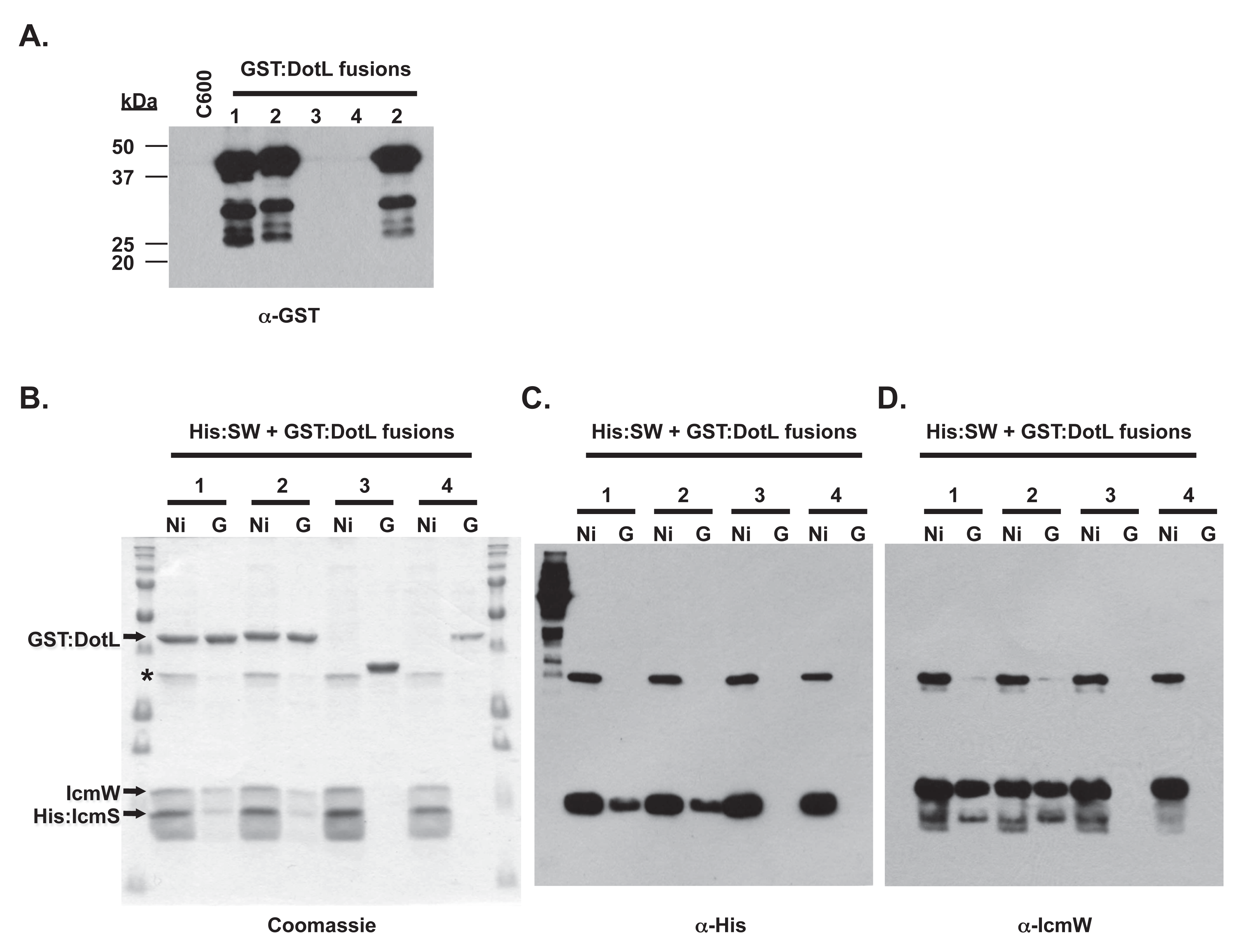

Supplement: Figure S7 — DotL and IcmSW directly interact. (A) Non-stoichiometric bands from the tandem affinity purification are GST:DotL degradation products. Samples used in Fig. 5 were separated on an SDS-PAGE gel, probed with a GST specific antibody, revealing several degradation products. Fragment 2 was also loaded on the right side of the gel as a control. (B–D) Lysates containing His:SW and the four fragments from Fig. 5 were purified on either a Ni-NTA column (Ni) or a glutathione sepharose column (G). Elutions were run on SDS-PAGE gel and stained with Coomassie (B), probed with anti-His antibody to detect His-IcmS (C) or probed with anti-IcmW antibody (D). His:SW is able to bind Ni-NTA in all cases and all 4 GST:DotL fragments are able to bind glutathione sepharose. GST:DotL fragments 1 & 2 co-purify from the Ni column, but fragments 3 & 4 do not. His:SW co-purifies from the glutathione sepharose column when using fragments 1 & 2 but not with fragments 3 & 4. The * indicates a His:SW dimer that was not denatured by the SDS-PAGE gel and can be detected in the westerns (C & D). (TIF) [file ppat.1002910.s007.tif]

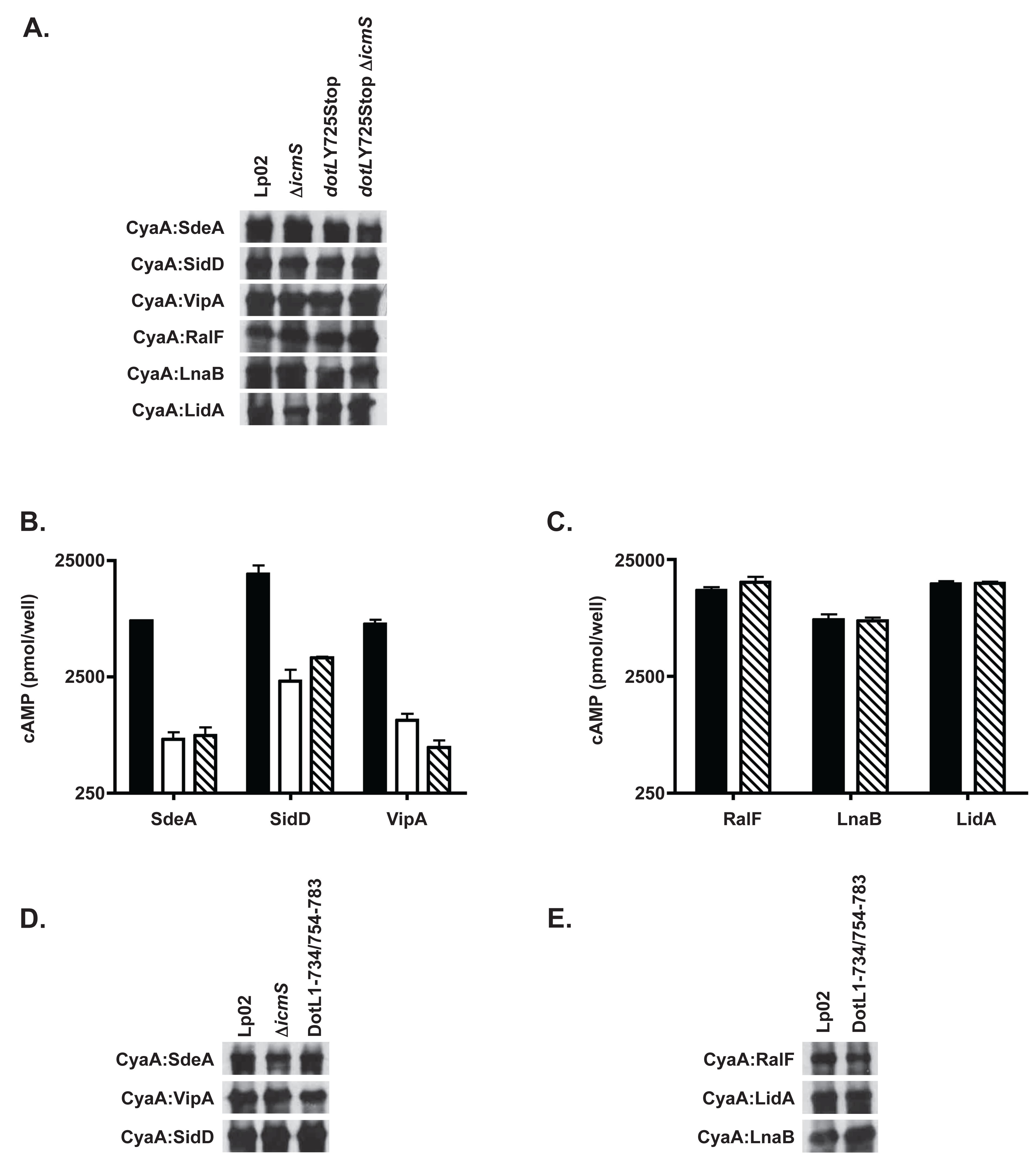

Supplement: Figure S8 — The DotL1–734/754–783 mutant is defective for secretion of only IcmSW-dependent substrates as observed for the original DotLY725Stop mutant. (A) Protein levels of CyaA fusions to Dot/Icm substrates used in Figure 7 were assessed by western blot with a CyaA-specific antibody and shown to produce equivalent levels of protein. (B & C) Export of CyaA fusions to IcmSW-dependent effectors (SdeA, SidD, VipA) and IcmSW-independent effectors (RalF, LnaB, LidA) were assayed in a wild-type strain (black bars), ΔicmS mutant (white bars), and a strain with DotL1–734/754–783 integrated onto the chromosome (striped bars). cAMP was measured from triplicate wells and error bars represent the standard deviation from the mean. (D & E) Protein samples from Fig. S8B and S8C were analyzed as in Fig. S8A. (TIF) [file ppat.1002910.s008.tif]
