# Supplementary material for: The Legionella IcmSW Complex Directly Interacts with DotL to Mediate Translocation of Adaptor-Dependent Substrates
Source: PLoS Pathog. 2012 Sep 13;8(9):e1002910. doi: 10.1371/journal.ppat.1002910 (PMC3441705; doi:10.1371/journal.ppat.1002910)
Supplement: Table S1 — dotL Y725Stop is able to transfer an RSF1010 mobilizable plasmid. Mobilization of the RSF1010 plasmid by the L. pneumophila T4SS to E. coli recipients was determined. The rate of transfer was calculated by dividing the average number of recipients by the average total number of donor cells. (PDF) [file ppat.1002910.s009.pdf]

Table S1. *dotL*Y725Stop is able to transfer an RSF1010 mobilizable plasmid

Description	Rate of Transfer
Wild type + RSF1010 *oriT*+	1.1E-5 +/- 3.1E-6
Wild type + RSF1010 *oriT*-	< 3.6E-8 +/- 4.8E-7
Δ*dotA* + RSF1010 *oriT*+	< 2.6E-8 +/- 4.2E-7
Δ*icmS* + RSF1010 *oriT*+	1.1E-5 +/- 3.6E-6
*dotL*Y725Stop + RSF1010 *oriT*+	2.1E-5 +/- 3.7E-6
*dotL*Y725Stop + RSF1010 *oriT*-	< 3.7E-8 +/- 5.2E-7
