# Supplementary material for: The Legionella IcmSW Complex Directly Interacts with DotL to Mediate Translocation of Adaptor-Dependent Substrates
Source: PLoS Pathog. 2012 Sep 13;8(9):e1002910. doi: 10.1371/journal.ppat.1002910 (PMC3441705; doi:10.1371/journal.ppat.1002910)
Supplement: Table S2 — IcmSW dependence of Dot/Icm substrates. (PDF) [file ppat.1002910.s010.pdf]

Table S2. IcmSW dependence of Dot/Icm substrates

Substrate	Lpg#	Type IV adaptor-dependent secretion?	Assay to determine type IV adaptor dependent secretion	Interaction with the type IV adaptors	Reference
AnkH	2300	No	5	ND	Habyarimana et al, 2010
AnkI	1718	No	5	ND	Habyarimana et al, 2010
AnkK	2322	No	5	ND	Habyarimana et al, 2010
AnkN	0695	No	5	ND	Habyarimana et al, 2010
LepA	2793	No	3	ND	Chen et al, 2007
LepB	2490	No	3	ND	Chen et al, 2007
LidA	0940	No	2	ND	Vincent et al, 2012
LnaB	2527	No	2	ND	This study
RalF	1950	No	1	No (6)	Nagai et al, 2005/Ninio et al, 2005/Cambronne & Roy, 2007
SidF	2584	No	1	ND	Cambronne & Roy, 2007
AnkD	2456	Yes	5	ND	Habyarimana et al, 2010
AnkG	0403	Yes	5	ND	Habyarimana et al, 2010
AnkJ	436	Yes	5	ND	Habyarimana et al, 2010
PieA	1963	Yes	4	ND	Ninio et al, 2009
PieB	1964	Yes	4	ND	Ninio et al, 2009
PieC	1965	Yes	4	ND	Ninio et al, 2009
PieD	1966	Yes	4	ND	Ninio et al, 2009
PieE	1969	Yes	4	ND	Ninio et al, 2009
PieF	1972	Yes	4	ND	Ninio et al, 2009
PieG	1976	Yes	4	ND	Ninio et al, 2009
PpeA	1701	Yes	4	ND	Ninio et al, 2009
PpeB	1702	Yes	4	ND	Ninio et al, 2009
PpgA	2224	Yes	4	ND	Ninio et al, 2009
SdeA	2157	Yes	1	Yes (7)	Coers et al, 2000/Bardill et al, 2005
SidA	0621	Yes	1	ND	Cambronne & Roy, 2007
SidB	1642	Yes	1	ND	Cambronne & Roy, 2007
SidC	2511	Yes	1	ND	Cambronne & Roy, 2007
SidD	2465	Yes	1	ND	Cambronne & Roy, 2007
SidE	0234	Yes	1	ND	Cambronne & Roy, 2007
SidG	1355	Yes	1, 4	Yes (8)	Ninio et al, 2005/Cambronne & Roy, 2007
SidH	2829	Yes	1	Yes (9)	Ninio et al, 2005
VipA	0390	Yes	2	ND	Vincent et al, 2012
WipA	2718	Yes	1	Yes (8)	Ninio et al, 2005
WipB	0642	Yes	1	Yes (9)	Ninio et al, 2005

Assay for type IV adaptor-dependent secretion:
1 - Adenylate cyclase assay using a Δ*icmS* & a Δ*icmW* strain
2 - Adenylate cyclase assay using a Δ*icmS* strain
3 - Adenylate cyclase assay using a Δ*icmS* strain (data not shown)
4 - Adenylate cyclase assay using a Δ*icmS* Δ*icmW* strain
5 - Adenylate cyclase assay - data not shown

Assay for interaction with the type IV adaptor proteins:
6 - co-affinity purification with His:IcmW+IcmS
7 - Far western & affinity chromatography with GST:IcmS
8 - Yeast two-hybrid with IcmW & co-affinity purification with His:IcmW+IcmS
9 - Yeast two-hybrid with IcmW
ND - Not determined
