# Supplementary material for: The Legionella IcmSW Complex Directly Interacts with DotL to Mediate Translocation of Adaptor-Dependent Substrates
Source: PLoS Pathog. 2012 Sep 13;8(9):e1002910. doi: 10.1371/journal.ppat.1002910 (PMC3441705; doi:10.1371/journal.ppat.1002910)
Supplement: Table S3 — Relevant strains, plasmids, and primers employed in this study. (PDF) [file ppat.1002910.s011.pdf]

Table S3. Relevant strains, plasmid, and primers employed in this study

Strain, Plasmid, Primer	Relevant properties	Reference or source
L. pneumophila		
Lp02	Philadelphia-1 *rspL hsdR thyA*	Berger and Isberg, 1993
Lp03	Lp02 *dotA*	Berger and Isberg, 1993
JV1003	Lp02 *dotL*/Δ*dotL*::Cm^R	Sexton et al, 2005
JV1138	Lp02 + pKB5	This study
JV1139	Lp02 + pJB908	Bardill et al, 2005
JV1141	Lp03 + pJB908	Bardill et al, 2005
JV1962	Lp02 Δ*icmS*	Vincent et al, 2006
JV2064	Lp02 Δ*dotA*	Vincent et al, 2006
JV2282	Lp02 Δ*icmS dotL*/Δ*dotL*::Cm^R	This study
JV2422	Lp02 Δ*dotA* Δ*dotL*::CmR	Buscher et al, 2005
JV2700	Lp02 + CyaA:SdeA (pJB2588)	Bardill et al, 2005
JV2700	Lp02 + CyaA:SdeA (pJB2588)	Bardill et al, 2005
JV3901	Lp02 + CyaA:RalF (pJB2590)	Bardill et al, 2005
JV4159	Lp02 Δ*dotA* + pKB5	This study
JV5705	Lp02 + CyaA:VipA (pJB4509)	This study
JV5810	Lp02 Δ*icmS* + pKB5	This study
JV6064	Lp02 Δ*icmS* (JV1962) + CyaA:RalF (pJB2590)	This study
JV6066	Lp02 Δ*icmS* (JV1962) + CyaA:SdeA (pJB2588)	This study
JV6069	Lp02 Δ*icmS* (JV1962) + CyaA:VipA (pJB4509)	This study
JV6688	Lp02 Δ*dotL*::CmR + DotL amino acids 1-773 (pJB5273)	This study
JV6698	Lp02 Δ*dotA* Δ*dotL*::Cm^R + pJB4858	This study
JV6743	Lp02 + CyaA:SidD (pJB5169)	This study
JV6867	Lp02 Δ*dotL*::Cm^R + DotLY531C (pJB5289)	This study
JV6963	Lp02 Δ*dotL*::Cm^R + DotLN393D (pJB5285)	This study
JV6964	Lp02 Δ*dotL*::Cm^R + DotLF460L (pJB5286)	This study
JV6965	Lp02 Δ*dotL*::Cm^R + DotLY725Stop (pJB5287)	This study
JV6966	Lp02 Δ*dotL*::Cm^R + DotLD533N (pJB5288)	This study
JV6969	Lp02 Δ*dotL*::Cm^R + DotLK386R (pJB5291)	This study
JV6970	Lp02 Δ*dotL*::Cm^R + DotLF275S (pJB5292)	This study
JV6971	Lp02 Δ*dotL*::Cm^R + DotLQ222R (pJB5293)	This study
JV6972	Lp02 Δ*dotL*::Cm^R + DotLN312D (pJB5294)	This study
JV6974	Lp02 Δ*dotL*::Cm^R + DotLM239T (pJB5296)	This study
JV6975	Lp02 Δ*dotL*::Cm^R + *dotL*+ (pJB4860)	This study
JV6996	Lp02 Δ*dotL*::Cm^R + DotLN486T (pJB5284)	This study
JV6997	Lp02 Δ*dotL*::Cm^R + DotLE390G (pJB5295)	This study
JV7283	Lp02 Δ*dotL*::Cm^R + DotL amino acids 1-694/7115-783 (pJB6320)	This study
JV7287	Lp02 Δ*icmS* Δ*dotL*::Cm^R + DotL amino acids 1-694/715-783 (pJB6320)	This study
JV7325	Lp02 DotLY725Stop	This study
JV7502	Lp02 DotLY725Stop (JV7325) + CyaA:SdeA (pJB2588)	This study
JV7503	Lp02 DotLY725Stop (JV7325) + CyaA:RalF (pJB2590)	This study
JV7506	Lp02 DotLY725Stop (JV7325) + CyaA:VipA (pJB4509)	This study
JV7584	Lp02 DotLY725Stop + JB908	This study
JV7585	Lp02 DotLY725Stop + pKB5	This study
JV7590	Lp02 Δ*dotA* Δ*dotL*::Cm^R + DotL amino acids 1-725 (pJB5499)	This study
JV7592	Lp02 Δ*dotA* Δ*dotL*::Cm^R + DotL amino acids 1-733 (pJB5556)	This study
JV7593	Lp02 Δ*dotA* Δ*dotL*::Cm^R + DotL amino acids 1-743 (pJB5557)	This study
JV7638	Lp02 Δ*dotA* Δ*dotL*::Cm^R + DotL amino acids 1-724/734-783 (pJB6021)	This study
JV7645	Lp02 DotLY725Stop Δ*icmS*	This study
JV7659	Lp02 + CyaA:LidA (pJB5797)	This study
JV7661	Lp02 DotLY725Stop (JV7325) + CyaA:LidA (pJB5797)	This study
JV7662	Lp02 Δ*icmS* (JV1962) + CyaA:LidA (pJB5797)	This study
JV7690	Lp02 DotLY725Stop Δ*icmS* (JV7645) + CyaA:SdeA (pJB2588)	This study
JV7691	Lp02 DotLY725Stop Δ*icmS* (JV7645) + CyaA:RalF (pJB2590)	This study

JV7694	Lp02 DotLY725Stop Δ*icmS* (JV7645) + CyaA:VipA (pJB4509)	This study
JV7699	Lp02 DotLY725Stop Δ*icmS* (JV7645) + CyaA:LidA (pJB5797)	This study
JV7717	Lp02 + CyaA:LnaB (pJB5562)	This study
JV7718	Lp02 Δ*dotA* Δ*dotL*::Cm^{R} + DotL amino acids 1-773 (pJB5273)	This study
JV7719	Lp02 Δ*dotA* Δ*dotL*::Cm^{R} + DotL amino acids 1-763 (pJB5275)	This study
JV7720	Lp02 Δ*dotA* Δ*dotL*::Cm^{R} + DotL amino acids 1-753 (pJB5277)	This study
JV7728	Lp02 Δ*icmS* (JV1962) + CyaA:SidD (pJB5169)	This study
JV7730	Lp02 Δ*icmS* (JV1962) + CyaA:LnaB (pJB5562)	This study
JV7732	Lp02 DotLY725Stop (JV7325) + CyaA:SidD (pJB5169)	This study
JV7734	Lp02 DotLY725Stop (JV7325) + CyaA:LnaB (pJB5562)	This study
JV7744	Lp02 DotLY725Stop ΔicmS (JV7645) + CyaA:SidD (pJB5169)	This study
JV7746	Lp02 DotLY725Stop ΔicmS (JV7645) + CyaA:LnaB (pJB5562)	This study
JV7761	Lp02 Δ*dotA* Δ*dotL*::Cm^{R} + *dotL*+ (pJB4860)	This study
JV7762	Lp02 Δ*dotA* Δ*dotL*::Cm^{R} + DotL amino acids 1-694/705-783 (pJB5751)	This study
JV7763	Lp02 Δ*dotA* Δ*dotL*::Cm^{R} + DotL amino acids 1-684/695-783 (pJB5752)	This study
JV7764	Lp02 Δ*dotA* Δ*dotL*::Cm^{R} + DotL amino acids 1-674/685-783 (pJB5753)	This study
JV7765	Lp02 Δ*dotA* Δ*dotL*::Cm^{R} + DotL amino acids 1-704/715-783 (pJB5754)	This study
JV7766	Lp02 Δ*dotA* Δ*dotL*::Cm^{R} + DotL amino acids 1-709/720-783 (pJB5755)	This study
JV7767	Lp02 Δ*dotA* Δ*dotL*::Cm^{R} + DotL amino acids 1-714/725-783 (pJB5756)	This study
JV7876	Lp02 Δ*dotA* Δ*dotL*::Cm^{R} + DotL amino acids 1-670/754-783 (pJB6101)	This study
JV7883	Lp02 Δ*dotA* Δ*dotL*::Cm^{R} + DotL amino acids 1-734/754-783 (pJB6108)	This study
JV7929	Lp02 Δ*dotA* Δ*dotL*::Cm^{R} + DotL amino acids 1-749/760-783 (pJB6129)	This study
JV7930	Lp02 Δ*dotA* Δ*dotL*::Cm^{R} + DotL amino acids 1-744/755-783 (pJB6130)	This study
JV7931	Lp02 Δ*dotA* Δ*dotL*::Cm^{R} + DotL amino acids 1-739/750-783 (pJB6131)	This study
JV7932	Lp02 Δ*dotA* Δ*dotL*::Cm^{R} + DotL amino acids 1-734/745-783 (pJB6132)	This study
JV7933	Lp02 Δ*dotA* Δ*dotL*::Cm^{R} + DotL amino acids 1-729/740-783 (pJB6133)	This study
JV7970	Lp02 Δ*dotA* Δ*dotL*::Cm^{R} + DotL amino acids 1-674/695-783 (pJB6319)	This study
JV7971	Lp02 Δ*dotA* Δ*dotL*::Cm^{R} + DotL amino acids 1-694/715-783 (pJB6320)	This study
JV7972	Lp02 Δ*dotA* Δ*dotL*::Cm^{R} + DotL amino acids 1-714/734-783 (pJB6321)	This study
JV7975	Lp02 DotL amino acids 1-735/754-783	This study
JV7992	Lp02 DotL amino acids 1-735/754-783 + CyaA:RalF (pJB2590)	This study
JV7993	Lp02 DotL amino acids 1-735/754-783 + CyaA:LidA (pJB5797)	This study
JV7994	Lp02 DotL amino acids 1-735/754-783 + CyaA:LnaB (pJB5562)	This study
JV7995	Lp02 DotL amino acids 1-735/754-783 + CyaA:SdeA (pJB2588)	This study
JV7996	Lp02 DotL amino acids 1-735/754-783 + CyaA:VipA (pJB4509)	This study
JV7997	Lp02 DotL amino acids 1-735/754-783 + CyaA:SidD (pJB5169)	This study
JV8117	Lp02 Δ*icmS* Δ*dotL*::Cm^{R} + *dotL* (pJB4860)	This study
JV8119	Lp02 Δ*icmS* Δ*dotL*::Cm^{R} + DotL amino acids 1-773 (pJB5273)	This study
E. coli		
C600	F- *thr leu thi lac tonA*	?
ER1821	F- glnV44 e14-(McrA-) rfbD1? relA1? endA1 spoT1? thi-1 Δ(mcrC-mrr)114::IS10	?
JB5470	C600 His:IcmS+IcmW (pJB5316) and GST:DotL amino acids 681-783 (pJB5424)	This study
JB5471	C600 His:IcmS+IcmW (pJB5316) and GST:DotL amino acids 671-783 (pJB5649)	This study
JB5472	C600 His:IcmS+IcmW (pJB5316) and GST:DotL amino acids 661-783 (pJB5647)	This study
JB5473	C600 His:IcmS+IcmW (pJB5316) and GST:DotL amino acids 651-783 (pJB5645)	This study
JB5474	C600 His:IcmS+IcmW (pJB5316) and GST:DotL amino acids 641-783 (pJB5643)	This study
JB5474	C600 His:IcmS+IcmW (pJB5316) and GST:DotL amino acids 641-783 (pJB5643)	This study
JB5475	C600 His:IcmS+IcmW (pJB5316) and GST:DotL amino acids 631-783 (pJB5641)	This study
JB5475	C600 His:IcmS+IcmW (pJB5316) and GST:DotL amino acids 631-783 (pJB5641)	This study
JB5564	C600 His:IcmS+IcmW (pJB5316) and GST:DotL amino acids 691-783 (pJB6092)	This study
JB5565	C600 His:IcmS+IcmW (pJB5316) and GST:DotL amino acids 671-780 (pJB6093)	This study
JB5566	C600 His:IcmS+IcmW (pJB5316) and GST:DotL amino acids 661-780 (pJB6094)	This study
JB5568	C600 His:IcmS+IcmW (pJB5316) and GST:DotL amino acids 671-753 (pJB6096)	This study
JB6204	C600 His:IcmS+IcmW (pJB5316) and GST:DotL amino acids 701-783 (pJB5582)	This study
JB6205	C600 His:IcmS+IcmW (pJB5316) and GST:DotL amino acids 711-783 (pJB5585)	This study
JB6214	C600 His:IcmS+IcmW (pJB5316) and GST:DotL amino acids 661-763 (pJB6202)	This study
JB6265	C600 His:IcmS+IcmW (pJB5316) and GST:DotL amino acids 631-670/754-783 (pJB5919)	This study

JB6266	C600 His:IcmS+IcmW (pJB5316) and GST:DotL amino acids 631-734/754-783 (pJB5921)	This study
JB6460	C600 His:IcmS+IcmW (pJB5316) and GST:DotL amino acids 661-773 (pJB6252)	This study
JB6461	C600 His:IcmS+IcmW (pJB5316) and GST:DotL amino acids 671-773 (pJB6254)	This study
JB6462	C600 His:IcmS+IcmW (pJB5316) and GST:DotL amino acids 671-763 (pJB6215)	This study
JB6463	C600 His:IcmS+IcmW (pJB5316) and GST:DotL amino acids 661-753 (pJB6256)	This study
JB6464	C600 pQE-30 Kan^{R} (pJB5457) and GST:DotL amino acids 631-783 (pJB5641)	This study
JB6465	C600 pQE-30 Kan^{R} (pJB5457) and GST:DotL amino acids 641-783 (pJB5643)	This study
JB6466	C600 pQE-30 Kan^{R} (pJB5457) and GST:DotL amino acids 651-783 (pJB5645)	This study
JB6467	C600 pQE-30 Kan^{R} (pJB5457) and GST:DotL amino acids 661-783 (pJB5647)	This study
JB6468	C600 pQE-30 Kan^{R} (pJB5457) and GST:DotL amino acids 671-783 (pJB5649)	This study
JB6469	C600 pQE-30 Kan^{R} (pJB5457) and GST:DotL amino acids 661-780 (pJB6094)	This study
JB6470	C600 pQE-30 Kan^{R} (pJB5457) and GST:DotL amino acids 671-780 (pJB6093)	This study
JB6471	C600 pQE-30 Kan^{R} (pJB5457) and GST:DotL amino acids 661-773 (pJB6252)	This study
JB6472	C600 pQE-30 Kan^{R} (pJB5457) and GST:DotL amino acids 671-773 (pJB6254)	This study
JB6473	C600 pQE-30 Kan^{R} (pJB5457) and GST:DotL amino acids 661-763 (pJB6202)	This study
Plasmid		
pKB5	RSF1010 cloning vector, thyA+, *bla*, *mob*	Berger and Isberg, 1993
pSR47S	R6K suicide vector (Kan^{R} *sacB*)	Merriam et al, 1997
pJB908	pKB5 Δ*oriT*	Sexton et al, 2004
pJB1005	pSR47S with *dotL* flanking regions and Cm^{R} replacing *dotL*	Buscher et al, 2005
pJB1455	pSR47S with *icmS* flanking regions	Vincent et al, 2006a
pJB2581	CyaA:X fusion vector RSF1010 Amp^{R} Cm^{R} Δ*oriT*	Bardill et al, 2005
pJB2588	CyaA:SdeA	Bardill et al, 2005
pJB2590	CyaA:RalF	This study
pJB3151	*dotL* complementing clone RSF1010 Amp^{R} Kan^{R} Δ*oriT*	This study
pJB4509	CyaA:VipA	Vincent et al, 2012
pJB4585	GST:X fusion vector RSF1010, Amp^{R}, tdΔi, Δ*oriT*	This study
pJB4858	RSF1010 cloning vector, Amp^{R}, tdΔi	This study
pJB5169	CyaA:SidD	This study
pJB5253	*dotL* complementing clone RSF1010 Amp^{R} tdΔi Δ*oriT*	This study
pJB5273	DotL amino acids 1-773	This study
pJB5275	DotL amino acids 1-763	This study
pJB5277	DotL amino acids 1-753	This study
pJB5284	DotL single amino acid point mutant: N486T	This study
pJB5285	DotL single amino acid point mutant: N393D	This study
pJB5286	DotL single amino acid point mutant: F460L	This study
pJB5287	DotL single amino acid point mutant: Y725Stop	This study
pJB5288	DotL single amino acid point mutant: D533N	This study
pJB5289	DotL single amino acid point mutant: Y531C	This study
pJB5291	DotL single amino acid point mutant: K386R	This study
pJB5292	DotL single amino acid point mutant: F275S	This study
pJB5293	DotL single amino acid point mutant: Q222R	This study
pJB5294	DotL single amino acid point mutant: N312D	This study
pJB5295	DotL single amino acid point mutant: E390G	This study
pJB5296	DotL single amino acid point mutant: M239T	This study
pJB5316	His:IcmS+IcmW ColE1 Kan^{R}	This study
pJB5424	GST:DotL amino acids 681-783	This study
pJB5457	pQE-30 ColE1 Kan^{R}	This study
pJB5499	DotL amino acids 1-725 with Shine-Dalgarno from T7	This study
pJB5556	DotL amino acids 1-733 with Shine-Dalgarno from T7	This study
pJB5557	DotL amino acids 1-743 with Shine-Dalgarno from T7	This study
pJB5562	CyaA:LnaB	This study
pJB5582	GST:DotL amino acids 701-783	This study
pJB5585	GST:DotL amino acids 711-783	This study
pJB5641	GST:DotL amino acids 631-783	This study
pJB5643	GST:DotL amino acids 641-783	This study
pJB5645	GST:DotL amino acids 651-783	This study

pJB5647	GST:DotL amino acids 661-783	This study
pJB5649	GST:DotL amino acids 671-783	This study
pJB5677	pSR47S with DotLY725Stop flanking regions	This study
pJB5738	*dotL* complementing clone with Shine-Dalgarno from T7 phage	This study
pJB5748	*dotL* (1-1629 bp) acceptor vector RSF1010 Amp^R Δ*oriT*	This study
pJB5751	DotL amino acids 1-694/705-783	This study
pJB5752	DotL amino acids 1-684/695-783	This study
pJB5753	DotL amino acids 1-674/685-783	This study
pJB5754	DotL amino acids 1-704/715-783	This study
pJB5755	DotL amino acids 1-709/720-783	This study
pJB5756	DotL amino acids 1-714/725-783	This study
pJB5797	CyaA:LidA	This study
pJB5919	GST:DotL amino acids 631-670/754-783	This study
pJB5921	GST:DotL amino acids 631-734/754-783	This study
pJB6021	DotL amino acids 1-724/734-783	This study
pJB6092	GST:DotL amino acids 691-783	This study
pJB6093	GST:DotL amino acids 671-780	This study
pJB6094	GST:DotL amino acids 661-780	This study
pJB6096	GST:DotL amino acids 671-753	This study
pJB6101	DotL amino acids 1-670/754-783	This study
pJB6108	DotL amino acids 1-734/754-783	This study
pJB6129	DotL amino acids 1-749/760-783	This study
pJB6130	DotL amino acids 1-744/755-783	This study
pJB6131	DotL amino acids 1-739/750-783	This study
pJB6132	DotL amino acids 1-734/745-783	This study
pJB6133	DotL amino acids 1-729/740-783	This study
pJB6202	GST:DotL amino acids 661-763	This study
pJB6215	GST:DotL amino acids 671-763	This study
pJB6252	GST:DotL amino acids 661-773	This study
pJB6254	GST:DotL amino acids 671-773	This study
pJB6256	GST:DotL amino acids 661-753	This study
pJB6319	DotL amino acids 1-674/695-783	This study
pJB6320	DotL amino acids 1-694/715-783	This study
pJB6321	DotL amino acids 1-714/734-783	This study
pJB6322	pSR47S with DotL amino acids 1-734/754-783 flanking regions	This study
Primer		
JVP1036	CAGGTGGGACCACCGCGC	
JVP1038	CCCGAATTCGGAATTAGAGCCATGATGCG	
JVP1039	CCCGCATGCCACTTCTACCTCCAATTGCCG	
JVP1070	CCCGTCGACTAATCATACATTAACTCATCC	
JVP1668	GCAGGATCCAAACACTATGATTCCATGGATCAGG	
JVP1669	GCAGTCGACGAACAGAAGTTAGCTTAACTAGAG	
JVP1702	CGCGAATTCGCATCTGTGCGGTATTTCACACC	
JVP1703	CGCGGATCCACGCGGAACCAGATCCGATTTTGG	
JVP1856	GCGAGCATGCTTCTAAAGTAAGTGTAATTGGCGGG	
JVP1857	GCGGCGGCCGCGCAGCAGCCTTAATGACAATAGTC	
JVP1986	CCAGGATCCCGTTCGATTATTACACAAATCTGTAATGG	
JVP1987	CCCGTCGACTTAAATAGTAAGACTCGAGTTAGTTGCAGCC	
JVP2020	CCATGTTGCTCCAAAACTTGGG	
JVP2022	CAAGGATTTGCGGTAGTGCCTGCGC	
JVP2022	CAAGGATTTGCGGTAGTGCCTGCG	
JVP2065	CCCGCATGCTTATTTCTCACGTTCTGCGGAAATTTTAGC	
JVP2065	CCCGCATGCTTATTTCTCACGTTCTGCGGAAATTTTAGC	
JVP2066	CCCGCATGCTTATAAATCTCTTATGATACCAGTAAGTTCTTG	
JVP2067	CCCGCATGCTTAGACATCAATCACATCCCTTTCTTCAGG	
JVP2112	CCCGGATCCCTACGAATTGACCCTAATGCTCCTCC	
JVP2112	CCCGGATCCCTACGAATTGACCCTAATGCTCCTCC	

JVP2113	CCCGTCGACCCGACTGAGTCTTTATGTTAATTCCTCTGC
JVP2145	CCCGTCGACGCAAACCGAAGGTGAGGCGC
JVP2146	CCCGGATCCCTATTTGTCTTTAGCCCCTGCCAAACGC
JVP2147	CCCGGATCCAGACTCAGTCGGCAATTGGAGG
JVP2157	CCCGCGGCCGCCAATACATAGATGTAGTTAGTACG
JVP2160	GTGCCATGGCCACGTTGTGTCTCAAAATCTCTG
JVP2161	GTGCCATGGGCCAGTGTTACAACCAATTAACC
JVP2178	CCCGGATCCATTAGTAAGGCATTAAAAGAATCTACTATCG
JVP2178	CCCGGATCCATTAGTAAGGCATTAAAAGAATCTACTATCG
JVP2179	CCCGGATCCGTAGAGCCAATTGAACGAGGCG
JVP2180	CCCGGATCCTTAATTGCATTTCATGGTCAAAATGAACC
JVP2181	CCCGGATCCGAGCCTGTGGAAGATATTGTAGAGG
JVP2181	CCCGGATCCGAGCCTGTGGAAGATATTGTAGAGG
JVP2182	CCCGGATCCGTTGAGGGGGCATTAACTATTTTCAGC
JVP2182	CCCGGATCCGTTGAGGGGGCATTAACTATTTTCAGC
JVP2244	CCCGGTACCTAAGAAGGAGGTATACATATGATGCGGGGTATTGATTCTCG
JVP2263	CCCGGTACCGTCAGCGACTAATATTGGAGGAGC
JVP2263	CCCGGTACCGTCAGCGACTAATATTGGAGGAGC
JVP2264	CCCGGTACCAATGAAACACGAAATCAAATGATAACG
JVP2265	CCCGGTACCGTCAATTCGTAGTTTGCTGAAAATAGTTAATGCC
JVP2266	CCCGGTACCAAAGAAGTTTTCTCTGAGCCATTATTACC
JVP2266	CCCGGTACCAAAGAAGTTTTCTCTGAGCCATTATTACC
JVP2267	CCCGGTACCTGCCCCCTCAACTTCTTCC
JVP2267	CCCGGTACCTGCCCCCTCAACTTCTTCC
JVP2268	CCCGGTACCCCTAATGCTCCTCCAATATTAGTCGC
JVP2269	CCCGGTACCAATTGGTAATAATGGCTCAGAGAAAACTTC
JVP2270	CCCGGTACCATTGAGCGTTTGGCAGGGGC
JVP2271	CCCGGTACCTCGTGTTTCATTAATTGGTAATAATGGC
JVP2272	CACGGTACCGGGGCTAAAGACAAATACGCTGG
JVP2273	CCCGGTACCCGTTATCATTTGATTTCGTGTTTCATTAATTGG
JVP2273	CCCGGTACCCGTTATCATTTGATTTCGTGTTTCATTAATTGG
JVP2274	CCCGGTACCTACGCTGGTACAGTAGCTAATG
JVP2279	CCCGGTACCGCTAATGAATTAATCAAAGATTTTCAAATAGCG
JVP2287	CCCGGTACCTTTGTCTTTAGCCCCTGCCAAACGC
JVP2289	CCCGGTACCATCAAAGATTTTCAAATAGCGACTAGC
JVP2289	CCCGGTACCATCAAAGATTTTCAAATAGCGACTAGC
JVP2293	CCCGTCGACCTAGACATCAATCACATCCCTTTCTTCAGG
JVP2318	CCCGGATCCTTAGTCGCTGACAAAGAAGTTTTCTCTGAGCC
JVP2319	CCCGTCGACCTACTCTGCAGCCTTTTTATTGGCTTTCTCACG
JVP2320	CCCGGTACCTTCTTCCTCTACAATATCTTCCACAGGC
JVP2323	CCCGGTACCGATTAATTCATTAGCTACTGTACCAGCG
JVP2325	GCAGGATCCTCATACCAAAAAATAGAACTCAGTATTTC
JVP2326	GCAGTCGACGCAAGAGATCTATTGTAAACGAGC
JVP2356	CCCGGATCCCCATTATTACCAATTAATGAAACACG
JVP2357	CCCGGATCCCAAATGATAACGATTGAGCGTTTGGC
JVP2366	CCCGTCGACCTATAAATCTCTTATGATACCAGTAAGTTCTTGG
JVP2367	CCCGGTACCTACTGTACCAGCGTATTTGTCTTTAGCCCC
JVP2368	CCCGGTACCGCGACTAGCTATCCTCCTGAAGAAAGGG
JVP2369	CCCGGTACCGATTAATTCATTAGCTACTGTACCAGCG
JVP2370	CCCGGTACCCCTGAAGAAAGGGATGTGATTGATGTCC
JVP2371	CCCGGTACCTATTTGAAAATCTTTGATTAATTCATTAGCTACTGTACCAGCG
JVP2372	CCCGGTACCGTGATTGATGTCCAAGAACTTACTGG
JVP2373	CCCGGTACCAGGATAGCTAGTCGCTATTTGAAAATCTTTGATTAATTCATTAGC
JVP2374	CCCGGTACCGAACTTACTGGTATCATAAGAGATTTATCAGC
JVP2375	CCCGGTACCATCCCTTTCTTCAGGAGGATAGCTAGTCGC
JVP2376	CCCGGTACCATAAGAGATTTATCAGCTAAAATTTCCGCAGAACG
JVP2392	CCCGTCGACCGTGCTCGTATGTTTTATGCCAATCC
JVP2393	CCCGGATCCCGACTGAGTCTTTATGTTAATTCCTCTG

JVP2394	CCCGGATCCGCAATTGGAGGTAGAAGTGTTCC
JVP2395	CCAGCGGCCGCCGAAGTGATCTCACCTGTTATCC
JVP2413	CCCGTCGACCTATTTCTCACGTTCTGCGGAAATTTTAGC
JVP2888	CCCGGTACCCAAGAACTTACTGGTATCATAAGAG
JVP429	CCCGTCGACCACGTTCATCATGGCTTGG
JVP432	CCCGCGGCCGCAGAAGCAAAACCTGATATGC
JVP554	CCCGGATCCGAGCGAGATATTAGCAAGTGTATGG
JVP779	CCCGGATCCGCAAAAGATAACAAATCACATCAAG
JVP780	CCCGTCGACTATCTTATGATGTCTTGAATGG

Relevant restriction sites are underlined
