# Supplementary material for: The Legionella IcmSW Complex Directly Interacts with DotL to Mediate Translocation of Adaptor-Dependent Substrates
Source: PLoS Pathog. 2012 Sep 13;8(9):e1002910. doi: 10.1371/journal.ppat.1002910 (PMC3441705; doi:10.1371/journal.ppat.1002910)
Supplement: Table S4 — Construction of plasmids employed in this study. (PDF) [file ppat.1002910.s012.pdf]

Table S4. Construction of plasmids employed in this study

Plasmid	Relevant Properties	Vector	Primers or Subclone	Restriction Enzymes
pJB3151	*dotL* complementing clone RSF1010 Amp^R Kan^R Δ*oriT*	pJB3147	JVP1038/JVP1039	EcoRI/SphI
pJB4585	GST:X fusion vector RSF1010, Amp^R, tdΔi, Δ*oriT*	* See below		
pJB4858	RSF1010 cloning vector, AmpR, tdΔi	* See below		
pJB5169	CyaA:SidD	pJB2581	JVP1986/JVP1987	BamHI/SalI
pJB5253	*dotL* complementing clone RSF1010 Amp^R tdΔi Δ*oriT*	pJB4858	pJB3151	EcoRI/SphI
pJB5273	DotL amino acids 1-773	pJB5253	JVP2022/JVP2065	SacI/SphI
pJB5275	DotL amino acids 1-763	pJB5253	JVP2022/JVP2066	SacI/SphI
pJB5277	DotL amino acids 1-753	pJB5253	JVP2022/JVP2067	SacI/SphI
pJB5284	DotL single amino acid point mutant: N486T	pJB4858	JVP1038/JVP1039	EcoRI/SphI
pJB5285	DotL single amino acid point mutant: N393D	pJB4858	JVP1038/JVP1039	EcoRI/SphI
pJB5286	DotL single amino acid point mutant: F460L	pJB4858	JVP1038/JVP1039	EcoRI/SphI
pJB5287	DotL single amino acid point mutant: Y725Stop	pJB4858	JVP1038/JVP1039	EcoRI/SphI
pJB5288	DotL single amino acid point mutant: D533N	pJB4858	JVP1038/JVP1039	EcoRI/SphI
pJB5289	DotL single amino acid point mutant: Y531C	pJB4858	JVP1038/JVP1039	EcoRI/SphI
pJB5291	DotL single amino acid point mutant: K386R	pJB4858	JVP1038/JVP1039	EcoRI/SphI
pJB5292	DotL single amino acid point mutant: F275S	pJB4858	JVP1038/JVP1039	EcoRI/SphI
pJB5293	DotL single amino acid point mutant: Q222R	pJB4858	JVP1038/JVP1039	EcoRI/SphI
pJB5294	DotL single amino acid point mutant: N312D	pJB4858	JVP1038/JVP1039	EcoRI/SphI
pJB5295	DotL single amino acid point mutant: E390G	pJB4858	JVP1038/JVP1039	EcoRI/SphI
pJB5296	DotL single amino acid point mutant: M239T	pJB4858	JVP1038/JVP1039	EcoRI/SphI
pJB5316	His:IcmS+IcmW ColE1 Kan^R	* See below		
pJB5424	GST:DotL amino acids 681-783	pJB4585	JVP2112/JVP2113	BamHI/SalI
pJB5457	pQE-30 ColE1 Kan^R	pQE-30	JVP2160/JVP2161	BspHI
pJB5499	DotL amino acids 1-725 with Shine-Dalgarno from T7 phage	pJB5738	pJB5287	HindIII/SphI
pJB5556	DotL amino acids 1-733 with Shine-Dalgarno from T7 phage	pJB5738	pJB5281	HindIII/SphI
pJB5557	DotL amino acids 1-743 with Shine-Dalgarno from T7 phage	pJB5738	pJB5279	HindIII/SphI
pJB5562	CyaA:LnaB	pJB2581	JVP2325/JVP2326	
pJB5582	GST:DotL amino acids 701-783	pJB4585	JVP2356/JVP2113	BamHI/SalI
pJB5585	GST:DotL amino acids 711-783	pJB4585	JVP2357/JVP2113	BamHI/SalI
pJB5641	GST:DotL amino acids 631-783	pJB4585	JVP2178/JVP2113	BamHI/SalI
pJB5643	GST:DotL amino acids 641-783	pJB4585	JVP2179/JVP2113	BamHI/SalI
pJB5645	GST:DotL amino acids 651-783	pJB4585	JVP2180/JVP2113	BamHI/SalI
pJB5647	GST:DotL amino acids 661-783	pJB4585	JVP2181/JVP2113	BamHI/SalI
pJB5649	GST:DotL amino acids 671-783	pJB4585	JVP2182/JVP2113	BamHI/SalI
pJB5677	pSR47S with *dotL*Y725Stop flanking regions	* See below		
pJB5738	*dotL* complementing clone with Shine-Dalgarno from T7 phage	pJB4860	JVP2244/JVP2020	KpnI/BamHI
pJB5748	*dotL* (1-1629 bp) acceptor vector RSF1010 Amp^R Δ*oriT*	* See below		
pJB5751	DotL amino acids 1-694/705-783	pJB5748	JVP2022/JVP2263 & JVP2264/JVP1036	SacI/NotI
pJB5752	DotL amino acids 1-684/695-783	pJB5748	JVP2022/JVP2265 & JVP2266/JVP1036	SacI/NotI
pJB5753	DotL amino acids 1-674/685-783	pJB5748	JVP2022/JVP2267 & JVP2268/JVP1036	SacI/NotI
pJB5754	DotL amino acids 1-704/715-783	pJB5748	JVP2022/JVP2269 & JVP2270/JVP1036	SacI/NotI
pJB5755	DotL amino acids 1-709/720-783	pJB5748	JVP2022/JVP2271 & JVP2272/JVP1036	SacI/NotI
pJB5756	DotL amino acids 1-714/725-783	pJB5748	JVP2022/JVP2273 & JVP2274/JVP1036	SacI/NotI
pJB5797	CyaA:LidA	pJB2581	JVP779/JVP780	BamHI/SalI
pJB5919	GST:DotL amino acids 631-670/754-783	pJB4585	JVP2178/JVP2113	BamHI/SalI
pJB5921	GST:DotL amino acids 631-734/754-783	pJB4585	JVP2178/JVP2113	BamHI/SalI
pJB6021	DotL amino acids 1-724/734-783	pJB5748	JVP2022/JVP2287 & JVP2289/JVP1036	SacI/NotI
pJB6092	GST:DotL amino acids 691-783	pJB4585	JVP2318/JVP2113	BamHI/SalI
pJB6093	GST:DotL amino acids 671-780	pJB4585	JVP2182/JVP2319	BamHI/SalI
pJB6094	GST:DotL amino acids 661-780	pJB4585	JVP2181/JVP2319	BamHI/SalI
pJB6096	GST:DotL amino acids 671-753	pJB4585	JVP2182/JVP2293	BamHI/SalI
pJB6101	DotL amino acids 1-670/754-783	pJB5748	JVP2022/JVP2320 & JVP2888/JVP1036	SacI/NotI
pJB6108	DotL amino acids 1-734/754-783	pJB5748	JVP2022/JVP2323 & JVP2288/JVP1036	SacI/NotI
pJB6129	DotL amino acids 1-749/760-783	pJB5748	JVP2022/JVP2375 & JVP2376/JVP1036	SacI/NotI
pJB6130	DotL amino acids 1-744/755-783	pJB5748	JVP2022/JVP2373 & JVP2374/JVP1036	SacI/NotI
pJB6131	DotL amino acids 1-739/750-783	pJB5748	JVP2022/JVP2371 & JVP2372/JVP1036	SacI/NotI
pJB6132	DotL amino acids 1-734/745-783	pJB5748	JVP2022/JVP2369 & JVP2370/JVP1036	SacI/NotI
pJB6133	DotL amino acids 1-729/740-783	pJB5748	JVP2022/JVP2367 & JVP2368/JVP1036	SacI/NotI
pJB6202	GST:DotL amino acids 661-763	pJB4585	JVP2181/JVP2366	BamHI/SalI
pJB6215	GST:DotL amino acids 671-763	pJB4585	JVP2182/JVP2366	BamHI/SalI
pJB6252	GST:DotL amino acids 661-773	pJB4585	JVP2181/JVP2413	BamHI/SalI
pJB6254	GST:DotL amino acids 671-773	pJB4585	JVP2182/JVP2413	BamHI/SalI
pJB6256	GST:DotL amino acids 661-753	pJB4585	JVP2181/JVP2293	BamHI/SalI
pJB6319	DotL amino acids 1-674/695-783	pJB5748	JVP2022/JVP2267 & JVP2266/JVP1036	SacI/NotI
pJB6320	DotL amino acids 1-694/715-783	pJB5748	JVP2022/JVP2263 & JVP2270/JVP1036	SacI/NotI
pJB6321	DotL amino acids 1-714/734-783	pJB5748	JVP2022/JVP2273 & JVP2289/JVP1036	SacI/NotI
pJB6322	pSR47S with DotL amino acids 1-734/754-783 flanking regions	pSR47S	JVP2392/JVP2393 & JVP2394/JVP2395	SalI/NotI

*Detailed description of specific plasmid constructions:

pJB4585 GST:X fusion vector RSF1010, Amp^R, tdΔi, Δ*oriT*
pKB5 was digested with EcoRI/HpaI to remove Ptac and tdΔi. tdΔi was cloned back into the EcoRI/HpaI sites of pKB5 in the reverse orientation, resulting in the promoter-less pKB5 derative, pJB98. Δ*oriT* was subcloned on a SfiI/BstZI7I fragment from pJB908 and cloned into the SfiI/BstZI7I sites of pJB98, resulting in pJB1343. JVP1702/JVP1703 were used to amplify LacIQ, Ptac, and GST from pGEX-2T (GE-Healthcare Life Sciences), this PCR product was digested with EcoRI/BamHI and cloned into the EcoRI/BamHI sites of pJB1343, resulting in pJB4585. This vector expresses GST at the same levels as pGEX-2T, but has an RSF1010 origin, allowing for expression in *L. pneumophila.*

pJB4858 RSF1010 cloning vector, Amp^R, tdΔi
pJB1301 (pJB908 with HindIII site replaced by a NotI site) was digested with EcoRI to remove tdΔi, plasmid was re-ligated resulting in pJB4850, then tdΔi was amplified with JVP1856/JVP1857 and cloned into the SphI/NotI sites, resulting in pJ4858.

pJB5316 His:IcmS+IcmW ColE1 Kan^R
IcmS was amplified from Lp02 genomic DNA using JVP554/1070. This PCR product was digested with BamHI/SalI and cloned into the BamHI/SalI sites of pQE-30 (Qiagen), resulting in pJB2894. IcmW was subcloned from pJB2872 (His:IcmS+IcmW in pQE-30) using SalI/PstI and cloned into the SalI/PstI sites of pJB2894, resulting in pJB3204. pJB3204 was digested with BspHI to remove Amp^R. Kan^R was subcloned from pKRB11 (Reece et al, 1995) using SmaI and cloned into the BspHI sites of pJB3204, resulting in pJB5316.

pJB5677 pSR47S with *dotL*Y725Stop flanking regions
The N-terminal 500 bp of flanking *dotL*Y725Stop DNA was amplified with JVP2145/JVP2146. This fragment was digested with SalI/BamHI and cloned into the SalI/BamHI sites of pSR47S, resulting in pJB5451. Next the C-terminal 500 bp of flanking *dotL*Y725Stop DNA was amplified with JVP2157/JVP2147. This fragment was digested with BamHI/NotI and cloned into the BamHI/NotI sites of pSR47S, resulting in pJB5468. The N-terminal 500 bp of flanking *dotL*Y725Stop was subcloned from pJB5451 and cloned into the SalI/BamHI sites of pJB5468, resulting pJB5677.

pJB5748 *dotL* (1-1629 bp) acceptor vector RSF1010 Amp^R Δ*oriT*
pJB5253 was digested with EcoRI/SacI to obtain basepairs 1-1629 of *dotL*. This fragment was cloned into the EcoRI/SacI sites of pJB4850, resulting in an *dotL* acceptor vector that contains the first 1629 bp that was used to construct internal *dotL* deletions.
